# A Comprehensive Review of the Role of the Microbiota–Gut–Brain Axis via Neuroinflammation: Advances and Therapeutic Implications for Ischemic Stroke

**DOI:** 10.3390/biom15070920

**Published:** 2025-06-23

**Authors:** Hui Guo, Xiang Tang, Xinyi He, Yizhen Weng, Quanquan Zhang, Qi Fang, Lulu Zhang

**Affiliations:** 1 Department of Neurology, First Affiliated Hospital of Soochow University, No. 899 Pinghai Road, Suzhou 215006, China; 20235232080@stu.suda.edu.cn (H.G.); tangxiang@suda.edu.cn (X.T.); 20235232075@stu.suda.edu.cn (X.H.); 20234232030@stu.suda.edu.cn (Y.W.); zhangquanquan@suda.edu.cn (Q.Z.); 2 Department of Neurology, Dushu Lake Hospital, Suzhou 215006, China

**Keywords:** ischemic stroke, microbiome–gut–brain axis, neuroinflammation, gut-derived metabolites, neurotransmitters, microglia

## Abstract

The human gastrointestinal tract harbors a complex and diverse microbial community. Emerging evidence has revealed bidirectional communication between the gut microbiome and the central nervous system, termed the “microbiota–gut–brain axis”. This axis serves as a critical regulator of glial cell function, positioning it as an essential target for ameliorating the onset and progression of ischemic stroke. In this review, we discuss the developments in the relationship between ischemic stroke and neuroinflammation via MGBA. The gut microbiome plays a critical role in signaling to microglia, astrocytes, and other immune components within this axis. We also summarize the interactions between the gut microbiota and glial cells under both healthy and ischemic stroke conditions. Additionally, we also focus on the role of microbiota-derived metabolites and neurotransmitters in ischemic stroke. Furthermore, we investigate the potential of targeting the intestinal and blood–brain barriers to improve MGBA. Finally, we evaluate the preclinical and clinical evidence for dietary interventions, probiotics, prebiotics, and fecal microbiota transplantation in ischemic stroke. A comprehensive understanding of the MGBA is essential for developing MGBA-based treatment for ischemic stroke.

## 1. Introduction

The CNS (central nervous system) and the GIT (gastrointestinal tract) are increasingly recognized for their intricate bidirectional interactions, which are mediated through chemical signals and mutual influences on the homeostasis of both systems. The MGBA (microbiota–gut–brain axis), a new concept, was introduced to explain the complex relationship between intestinal microbiota and the host [1]. Recent studies have underscored the critical role of the gut microbiota in this axis, particularly in the pathophysiology of neurological and psychiatric disorders [2].

As one of the most prevalent neurological disorders, stroke remains a leading cause of disability and mortality worldwide, imposing a considerable burden on individuals, families, and healthcare systems [3,4]. Ischemic stroke occurs due to brain tissue necrosis caused by insufficient or interrupted blood flow. The gut microbiota, along with its metabolites, plays a pivotal role in the pathophysiology of ischemic stroke, primarily through inflammatory mechanisms. Nearly half of stroke patients develop gastrointestinal complications, such as motility disorders, dysphagia, fecal incontinence, leaky gut, intestinal bleeding, and, in severe cases, enterogenic sepsis [5]. Studies indicate that stroke patients with severe gut dysbiosis may experience poorer neurological outcomes [6], suggesting a potential role of the gut microbiota in influencing stroke recovery in humans [7,8].

Following a stroke, intestinal microbial dysbiosis triggers an increase in intestinal permeability and activation of the intestinal immune system. This disruption enables the entry of ectopic intestinal bacteria and pro-inflammatory cells into the brain tissue via the compromised BBB (blood–brain barrier), exacerbating ischemia–reperfusion injury [8,9]. Notably, certain metabolites produced by the gut microbiota after a stroke have been shown to attenuate ischemia–reperfusion injury by modulating the post-stroke inflammatory response and promoting neurological repair. In this review, we elucidate the changes in the gut flora at various stages after ischemic stroke, providing a comprehensive overview of recent research advances into the MGBA and its involvement in AIS (acute ischemic stroke), with a particular focus on the inflammatory and immune responses following a stroke.

### Microbiome–Gut–Brain Axis

**Figure 1:**
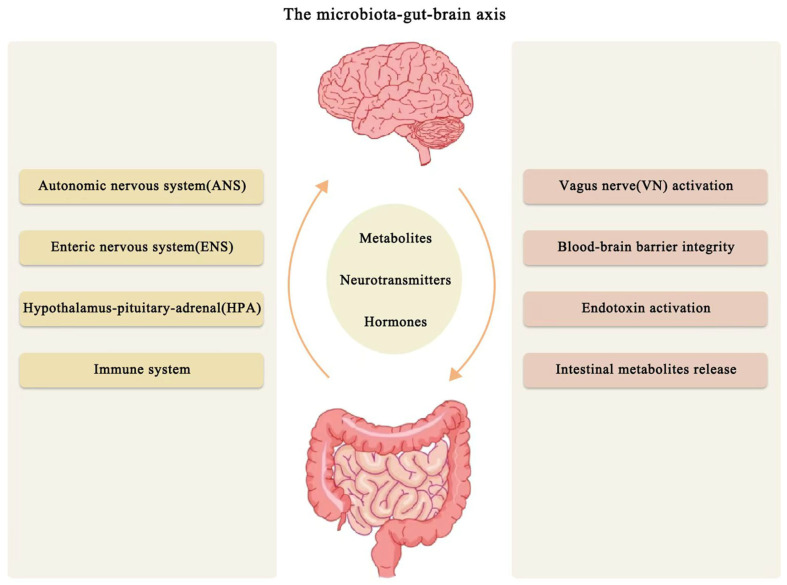
The microbiota–gut–brain axis. The bidirectional communication between the gut microbiome and the brain occurs via gut metabolites, neurotransmitters, and hormones, involving the ANS, enteric nervous system, HPA axis, and immune system. Changes in gut microbiota can activate the vagus nerve, affect BBB integrity, trigger endotoxin responses, and release intestinal metabolites.

The concept of the MGBA was first introduced by Sudo et al. in 2004 [10], a communication network encompassing the gut, nervous system (central, autonomic, and enteric), HPA (hypothalamic–pituitary–adrenal) axis, the neuroendocrine system, the immune system, gut microbiota, intestinal mucosal barrier, and the BBB [11]. This concept underlies the dynamic bidirectional communications that exist between the CNS and the GIT, functioning through three main pathways: immune, neuronal (including the sympathetic, parasympathetic, and enteric nervous systems), and endocrine (involving the HPA axis) [12]. These pathways facilitate dynamic interactions among gut microbiota, gut metabolism, and the CNS, ultimately influencing brain function (Figure 1). A crucial aspect of this axis is the bidirectional communication system that links the gut microbiota and the brain. The CNS transmits signals to the gut, regulating functions such as peristalsis, mucus secretion, and the mucosal immune system, influencing the diversity of gut bacteria through the ANS (autonomic nervous system). On the other hand, gut bacteria communicate with the CNS and immune system by releasing neurotransmitters, metabolites, and immune mediators, which can either ameliorate or exacerbate brain-related conditions [13,14].

There are unique microbiomes in almost every ecological niche of our body, with the major sites of colonization being the skin, respiratory tract, genitourinary tract, eyes, and GIT [15]. Significant advancements in sequencing technologies and the development of microbiome bioinformatics tools have made it increasingly affordable to analyze microbiota composition. To date, 2172 species have been identified across the human bodies, including 12 different phyla, with 386 species of anaerobic bacteria thriving in the mucosal regions, such as the GIT and oral cavity [16]. While the oral and pulmonary microbiota are critical, the majority of our microbial residents reside in the gut, which houses a diverse array of microorganisms, including yeasts, archaea, helminths, viruses, protozoa, and bacteria—the latter has been the most extensively studied [15].

In humans, these microorganisms serve diverse functions, such as synthesizing vitamins B and K, producing derivatives such as SCFAs (short-chain fatty acids), bile acids, TMAO (trimethylamine N-oxide), LPS (lipopolysaccharides), and phenylacetylglutamine. Moreover, they are instrumental in metabolizing key compounds like amino acids (e.g., glycine, glutamic acid, Gamma-Aminobutyric Acid (GABA), aspartic acid), peptides (e.g., vasopressin, somatostatin, neurotensin), biogenic amines (e.g., norepinephrine, serotonin, dopamine), and ACh (Acetylcholine) [16]. They also ferment undigested carbohydrates and defend against pathogens. Ideally, the bacterial community residing in the gut should have evolved to function in a symbiotic manner with the host, facilitating digestion and fostering an appropriate immune response.

However, dysbiosis of the gut can lead to excessive bacterial growth in the intestinal region, resulting in an increase in the permeability barrier and the induction of systemic inflammation. This inflammatory cascade overactivates the immune system, setting the stage for the development of various diseases [17]. A notable clinical example of this is hepatic encephalopathy, where targeting the microbiota with antibiotics can help reduce ammonia production in the gut, a key contributor to this disease [18]. By altering the gut microbiome, antibiotics may improve cognitive function and overall outcomes in patients suffering from this condition, and this also synergizes with the concept of the microbiome-gut–brain axis.

So far, the MGBA has been implicated in a wide range of diseases, including neurodegenerative conditions such as Alzheimer’s disease, Parkinson’s disease, and ALS (amyotrophic lateral sclerosis). It also plays a role in ischemic stroke, depression, autism spectrum disorder, dementia, mild cognitive impairment, multiple sclerosis, inflammatory bowel disease, drug-resistant epilepsy, and insomnia. These diverse conditions underscore the importance of understanding the MGBA as a potential therapeutic target for a variety of neurological and systemic disorders [19].

## 2. Pathophysiology of Acute Ischemic Stroke

AIS occurs when there is a severe reduction in cerebral blood flow, leading to focal hypoperfusion in the affected region. This oxygen and nutrient deprivation lead to neuronal cell death and irreversible tissue damage, known as the core infarction. Surrounding this core is the penumbra, an area of tissue with markedly reduced blood flow that still maintains viability above the threshold for cell death. This penumbral tissue remains vulnerable and can survive for several hours, making timely reperfusion critical to rescue these cells and prevent further damage to the brain [20,21].

With the advancement of modern technology, imaging techniques like computed tomography perfusion or MRI, which play a crucial role in identifying salvageable penumbral tissue, guide decisions on reperfusion therapies such as tissue plasminogen activators and mechanical thrombectomy, which are designed to restore blood flow and minimize ischemic damage in the acute phase of ischemic stroke [22,23,24].

In addition, the initial ischemic event can also trigger the onset of secondary brain injury mechanisms. These mechanisms evolve over time and include oxidative stress, excitotoxicity, calcium dysregulation, cortical spreading depression, BBB disruption, cerebral edema, and neuroinflammation, among others [25]. Among these factors, neuroinflammation is particularly pivotal, as it can either lead to cell death and exacerbate brain damage or aid in neurological recovery. During these processes, pro-inflammatory signals generated by immune mediators rapidly activate resident cells, such as microglia and astrocytes, and promote the infiltration of various inflammatory cells (e.g., neutrophils, monocytes/macrophages, and T cells) into the ischemic area [26]. This inflammatory response, while initially protective, can become maladaptive if unchecked, worsening the extent of tissue injury and hindering recovery. The intricate balance between beneficial and detrimental inflammation is therefore crucial in determining the trajectory of recovery following an ischemic stroke.

## 3. Inflammation and Immune Response After Ischemic Stroke

In AIS, the deprivation of oxygen and nutrients triggers a cascade of inflammatory responses that exacerbate brain injury and disrupt the integrity of the BBB. The breakdown of the BBB during the early stages of ischemic stroke allows intestinal bacteria and their byproducts to enter the bloodstream, initiating systemic inflammation. This systemic inflammation further exacerbates brain damage and impairs neural function recovery following a stroke. Moreover, the dysregulation of the gut microbiota due to stroke can impact overall health and directly worsen neurological outcomes. Notably, the crosstalk between the brain and the gut plays a significant role in the pathophysiology of ischemic stroke onset, progression, and recovery, particularly through neuroimmune interactions. In this chapter, we discussed several key immune cells in both the CNS and the GIT, which engage in the whole process of post-stroke inflammatory response (Table 1).

### 3.1. Role of Microglia in Ischemic Stroke

**Figure 2:**
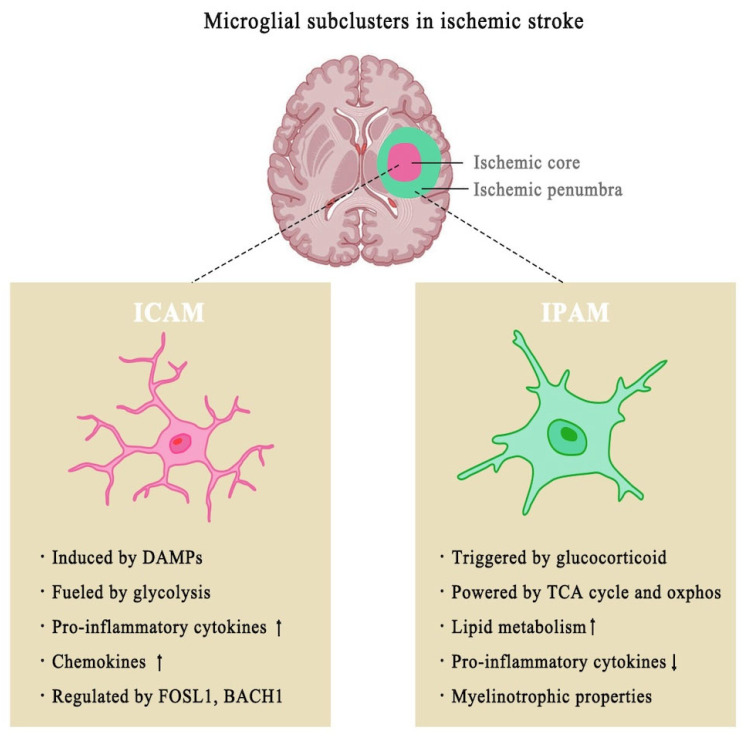
Microglia clusters in ischemic stroke. ① ICAM locate at the core of the inferction area, and are triggered by DAMPs (damage-associated molecular patterns) and powered by glycolysis. They act as a pro-inflammatory catalyst, summoning peripheral immune cells to further infiltrate the CNS, intensifying cerebral ischemic injury in its acute stage. ② IPAM surrounding around the ischemic lesion, fueled by the TCA cycle and oxidative phosphorylation, boast anti-inflammatory metabolic properties and potential neuro- and myelin-protective effects.

When discussing immune cells within the CNS, microglia inevitably warrant particular emphasis. Microglia—the resident immune cells of the CNS—undergo dynamic functional and phenotypic transformations in response to brain injury [27]. These changes are particularly well-characterized in the Middle Cerebral Artery Occlusion (MCAO) model, which serves as the gold standard experimental paradigm for ischemic stroke research in rodents [28]. This model recapitulates the key pathophysiological features of human stroke through surgical occlusion of the middle cerebral artery (MCA), with both transient (tMCAO) and permanent (pMCAO) variants allowing for an investigation of different ischemic durations. Under homeostatic conditions, microglia perform a variety of essential physiological functions, including regulating neurogenesis and angiogenesis, maintaining BBB integrity, synaptic pruning and remodeling, facilitating synaptic transmission, supporting myelin health, and engaging in the phagocytosis and clearance of apoptotic neurons and debris [29]. Notably, a recent single-cell RNA sequencing study revealed two distinct microglial subclusters that exhibit differential activation patterns under MCAO-induced ischemic conditions [30]. These two subclusters located in different regions of the ischemic brain: ICAM (ischemic core-associated microglia) in the infarcted area and IPAM (ischemic penumbra-associated microglia) in the surrounding penumbra [30]. These subclusters exhibit distinct functional and metabolic profiles, as demonstrated by GSVA (gene set variation analysis) (Figure 2). It was found that ICAM primarily relies on glycolysis, suggesting that they shift towards anaerobic metabolic pathways in response to ischemic injury. IPAM depend on the TCA (tricarboxylic acid) cycle and oxidative phosphorylation, indicative of a more oxidative metabolic profile. This metabolic divergence is linked to their distinct roles in stroke pathology. ICAM are associated with excessive pro-inflammatory responses and enhanced chemotaxis, suggesting they may contribute to tissue damage and exacerbate the acute inflammatory response during ischemic stroke. Their hyperactivation could accelerate neuronal injury and worsen stroke outcomes.

In comparison, IPAM display enriched metabolic pathways related to amino acids, lipids, and carbohydrates. Since lipid metabolites are essential components of myelin, IPAM may compensate for the loss of oligodendrocyte function by supplying lipid components. The role of lipid metabolites in neuroinflammation has been well-documented, and the anti-inflammatory properties of sphingolipid signaling are of increasing interest in neurodegenerative and neuroinflammatory research. The balance between these microglial subtypes and their metabolic pathways may therefore be a critical factor in determining stroke outcomes, influencing both the progression of neuroinflammation and the potential for recovery.

### 3.2. Microglial Activation and Its Dual Role in Post-Ischemic Stroke Inflammation

**Figure 3:**
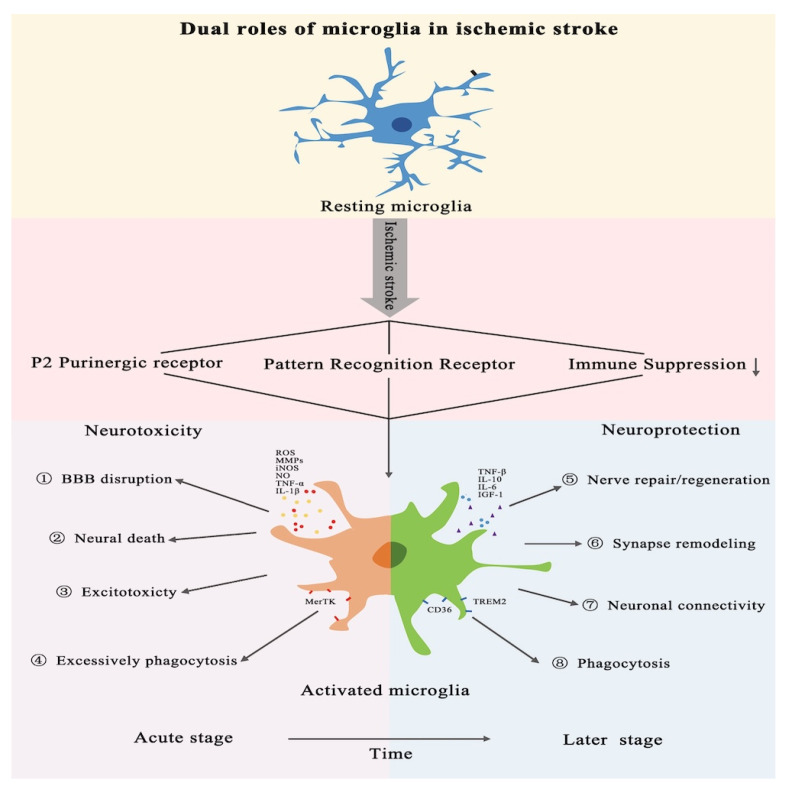
Dual roles of microglia in ischemic stroke. Upon the onset of ischemic stroke, quiescent microglial cells are activated in three ways. DAMPs bind to both P2PRs (P2XRs, P2YRs) and pattern recognition receptors (PRRs) (TLRs, NLRs, CLRs) on dormant microglia, inducing microglia activation. Additionally, post-stroke neuronal death leads to loss of immunosuppressants (CD200) and Chemokine (CX3CL1) on the neural surface, augmenting microglial activation. Acute stage: Microglia activates and migrates to the infarct area, exhibiting neurotoxic influences and worsening post-stroke neuroinflammation. ① Pro-inflammatory mediators released by microglia increase the BBB permeability. ② Overactivated microglia accelerate neural death. ③ Excitotoxicity is caused by excessive levels of glutamate and inflammatory chemokines. ④ Excessive phagocytosis: Surviving damaged neurons are identified by MFG-E8 (milk fat globule EGF factor 8) and combine with MerTK (Mer tyrosine kinase), inducing phagocytic cell death. Later stage: ⑤ Anti-inflammatory cytokines aid in neural repair and regeneration. ⑥ Synaptic remodeling is initiated by IL-10 via the JAK1/STAT3 signaling pathway. ⑦ Both IL-6 and IGF-1 contribute to the increasing neuronal connectivity through re-establishment of the neural network. ⑧ CD36 and TREM2 (triggering receptor expressed on myeloid cells 2) are primary phagocyte receptors on macrophages, facilitating microglial phagocytosis of post-stroke debris and DAMPs.

Microglial cells exhibit distinct functional states at different stages following ischemic stroke, positioning them as key players with dual roles in neuro-injury and neuroprotection (Figure 3). Early neuroinflammation, driven by microglial activation, serves as an adaptive microglial mechanism, providing neuroprotective effects by facilitating tissue repair, clearing cellular debris, and eliminating pathogens.

Following ischemic stroke, DAMPs are released, activating the PRRs on microglia. Additionally, accumulated ATP (adenosine triphosphate) and UDP (uridine triphosphate) in the ischemic tissue can activate P2 purinergic receptors on microglia. These events, coupled with the loss of immunosuppressants and chemokines from the neuronal surface, amplify microglial activation, leading to a vicious cycle of overactivation. This hyperactivity of microglia contributes to increased neuronal death and intensifies neuroinflammation, worsening the overall brain injury. In the late stage of AIS, activated microglia adopt a neuroprotective role, helping to mitigate excessive inflammation and restore homeostasis within the brain. Also, as the primary phagocytic cells in brain tissue, microglia can quickly clear cellular debris, reduce DAMPs, and protect the brain from secondary inflammatory response damage, promoting the recovery of nerve function and the re-establishment of the neural network.

Upon the onset of an ischemic stroke, quiescent microglia are rapidly activated and move towards the M1 subtype, initiating an inflammatory cascade characterized by the secretion of various cytokines and chemokines. This M1-driven inflammatory response disrupts the BBB and contributes to neuronal death. Conversely, microglia can also shift to the M2 subtype, which exhibits neuroprotective properties and secretes anti-inflammatory factors to facilitate tissue regeneration and repair [31]. The M1 subtype has been implicated in the generation of Th1 cells, with M1-polarized macrophages mediating the differentiation of helper T cells into Th1 and Th17 subsets. Given its pro-inflammatory nature, the M1 subtype can replace the M2 subtype, thereby perpetuating the inflammatory cycle.

The expression of major histocompatibility MHC-II (complex class II), along with CD16/32, CD40, and CD86 on the surface of M1 microglia, further stimulates the production of pro-inflammatory cytokines, such as TNF-α, IL-1β, IL-6, IL-12, and IL-23. Furthermore, microglia gradually lose their homeostatic signatures and become activated with age or in pathological conditions, adopting disease-associated phenotypes that release pro-inflammatory cytokines and chemokines [32]. Several genetically distinct microglial subtypes have been identified, including homeostatic microglia and “disease-associated microglia” (DAM) or the “microglial neurodegenerative phenotype”. Therefore, targeting the M1/M2 phenotypic switch of microglia or the DAM and the microglial neurodegenerative phenotype in the post-stroke period holds great promise as a therapeutic strategy to modulate neuroinflammation and promote recovery [33].

Given this, numerous clinical and laboratory studies have focused on exploring targets for intervention in microglial differentiation. Emerging evidence indicates that different dietary fibers can significantly modify the gut microbiome’s composition and function in obese mice on a high-fat diet [34,35]. This intervention is crucial for improving microglial cell maturation, which is essential for optimal brain function and health [36]. In addition to regulating microglial homeostasis, metabolites from gut microbes play a crucial role in triggering microglial cell death. Elevated concentrations of metabolites produced by gut microbes can cross the BBB, triggering programmed cell death in microglial cells. This process, which becomes more pronounced with advancing age, may contribute to the neuroinflammatory milieu observed in neurodegenerative diseases.

Among the gut-derived metabolites, SCFAs warrant particular attention due to their indispensable role in regulating microglial differentiation. SCFAs are important metabolic by-products of fiber digestion by gut bacteria, including acetate, propionate, and butyrate, which influence microglial behavior and function, ensuring proper development and activation in response to various signals from the body. The ability of the gut microbiome to influence microglial activation highlights the importance of the gut–brain axis in maintaining neural health. In certain pathological conditions, impaired microglial phagocytosis can result in the accumulation of toxic compounds, and excessive microglial activity may lead to neuronal loss and neurodegeneration through the phagocytosis of neurons [37].

In MCAO mouse models, supplementation with SCFAs significantly improved the recovery of motor function in the affected limb [38]. This improvement is believed to be mediated by the modulation of microglial activity. Following ischemic stroke, the intestinal flora activates microglia by producing endogenous AHR (aryl hydrocarbon receptor) ligands and SCFAs, which exert neuroprotective effects. The gut flora metabolizes tryptophan into AHR ligand-forming metabolites, which modulate microglial activation in neuroinflammatory conditions through their action on the microglial AHR [39,40]. This signaling pathway plays a pivotal role in the neuroprotective effects observed in the context of stroke and offers a potential therapeutic avenue for mitigating neuroinflammation and promoting recovery after ischemic events.

While gut-derived metabolites (e.g., SCFAs) have been the focus, emerging evidence underscores the indispensable roles of ApoE (Apolipoprotein E) and T cells in modulating microglial differentiation. Recent findings have clarified the complex interactions among microglia, ApoE, and T cells in neurological diseases [41]. ApoE, a lipid and cholesterol transporter, plays multiple roles in the CNS, including the regulation of microglial and astrocytic function, the maintenance of cerebrovascular and BBB integrity, myelin dynamics, and the modulation of neuronal network activity. Furthermore, T-cell infiltration into the CNS is critical for the activation of microglia and astrocytes in animal models of ALS [42]. In these models, treatment with natalizumab, which reduces immune cell recruitment, resulted in a significant reduction in pathological changes within the CNS, including decreased motor neuron degeneration, delayed onset of paralysis, and increased survival. These findings highlight the potential therapeutic benefit of targeting the signaling pathways between microglia and T cells to modulate neuroinflammation and improve outcomes in neurodegenerative diseases.

### 3.3. Other Immune Participants in Ischemic Stroke

The GIT is a key immune organ in the body, containing approximately 70% of immune cells [43], including a diverse array of immune cell types, such as B cells, Treg (regulatory T cells), macrophages, dendritic cells, γδT cells, αβT cells, and others. The intestinal mucosal immune system can be divided into three parts involved in defense: the columnar epithelial cell layer, the lamina propria, and the gut-associated lymphoid tissue. The intestinal mucosal immune defense comprises two distinct categories of sites: the immune effector sites and the immune inductive sites [43]. These sites collectively regulate a vast array of gut bacteria, maintaining a balance between tolerance of commensal microorganisms and effective immune regulation [44].

Inflammation and immunity play a crucial role in the pathophysiology of ischemic stroke, influencing all stages of the disease, from the development of risk factors to neurotoxicity and tissue remodeling during recovery [45,46]. Experimental research using rodent models has underscored the critical role of the immune system in stroke, particularly the brain-infiltrating lymphocytes originating from the intestinal immune compartment. These lymphocytes facilitate communication along the gut–brain axis, a key process in the neuroinflammatory response following ischemic injury [47,48,49,50].

The neuroinflammatory response in the brain is triggered and sustained by various components of the ischemic injury process, including necrotic cells, debris, and ROS (reactive oxygen species) [51]. This response involves the activation of resident inflammatory cells, the release of inflammatory mediators, and the migration of leukocytes across the BBB [52]. This, in turn, activates T and B lymphocytes, initiating an adaptive immune response [53]. In vivo cell-tracking studies, such as those conducted by V. Singh, have demonstrated the migration of intestinal lymphocytes to the ischemic brain, highlighting the integral role of the gut in modulating the immune response in stroke [54].

The neuroinflammatory cascade following ischemic injury is initiated by necrotic and apoptotic cells releasing DAMPs (e.g., HMGB1, ATP), which activate microglia through TLR4 (Toll-like receptor 4)/NLRP3 pathways [46]. This process is exacerbated by ROS: while the ischemic core shows sustained, high-level ROS (O_2_^−^, H_2_O_2_) due to mitochondrial collapse (Complex I/III inhibition and mPTP opening), the penumbra exhibits moderate, partially reversible ROS increases, mediated by the Nrf2/ARE antioxidant response [45,55]. In reaction to ischemic injury, necrotic cells, debris, and ROS participate in a second phase of immune activation—neuroinflammation, during which the neutrophils, microglia or macrophages, mast cells, lymphocytes, and NK T-cells are mobilized to the site of injury to initiate the neuroimmune response [56]. The activation of PRRs on these immune cells triggers the production of pro-inflammatory cytokines, such as IL-1β and TNF-α, which create an inflammatory environment, characterized by an increase in IL-17, perforin, granzyme and reactive oxidants, contributing to the worsening of tissue damage [57].

The necrotic tissues and brain cells release CNS-specific cryptic antigens, which can be recognized by APCs (antigen-presenting cells) in the brain. These APCs then migrate through the meningeal lymphatics into the peripheral circulation, eventually reaching the lymphoid organs, where they promote the differentiation of T and B cells. These differentiated self-reactive lymphocytes return to the brain tissue, potentially contributing to post-stroke sequelae, including chronic neuroinflammation and further neuronal injury [58].

T cell subsets play pivotal roles in this process, including both helper T cells (Th) and Treg. Both CD4+ and CD8+ T cell counts significantly increase after stroke, correlating with poorer functional outcomes. CD4+ T cells play a crucial role in the adaptive immune response within the intestine and can differentiate into various subtypes, including Th1, Th2, Th17 cells, and Tregs. Pro-inflammatory subsets, specifically Th1 and Th17 cells, can promote neuroinflammation [53,59]. Th1 cells exacerbate neuroinflammation and the activation of microglia by secreting cytokines such as IL-2, IL-12, and IFN-γ (Zhang et al., 2024 [59]). Th17 cells, on the other hand, can activate matrix metalloproteinases and contribute to the destruction of the BBB structure by producing cytokines such as IL-17A, IL-17F, and IL-22 [2,53,60,61].

Notably, the cytokine profiles also differ regionally (Table 2): the core is dominated by pro-inflammatory TNF-α and IL-1β from dying neurons and activated microglia, whereas the penumbra shows mixed signals, including anti-inflammatory IL-10 and TGF-β from infiltrating Tregs. Mitochondrial dysfunction plays a central role, with macrophages’ depolarization impairing ATP synthesis (leading to excitotoxicity) and released mtDNA fueling cGAS-STING-mediated neuroinflammation [62].

## 4. The Microbiome–Gut–Brain Axis in Ischemic Stroke

Multiple animal experiments and clinical evidence now suggest that there is bidirectional communication and interaction between the post-stroke brain and the GIT [59,63,64,65]. Two studies by Kaiyu Xu and colleagues explored post-stroke gut dysbiosis and its connection to stroke outcomes. They found significant differences in gut microbiota between stroke patients and healthy controls during the acute and subacute phases, with these disparities resolving during the recovery phase. Using an MCAO model in mice, they confirmed a link between gut dysbiosis and poor stroke outcomes [66]. However, the precise changes in the gut microbiota during post-stroke recovery remain poorly understood [67].

Several mechanisms, including glutamate excitotoxicity, calcium overload, neuroinflammation, oxidative stress, mitochondrial damage, and apoptosis, have been identified as contributors to the development of stroke [56]. A comprehensive analysis by Han et al., which reviewed 660 papers published between 2002 and 2021, explored the co-occurrence of stroke and gut microbiota [2]. They discovered that gut microbes can impact stroke by influencing metabolism, inflammation, and cardiovascular health. For instance, microbiota-derived SCFAs (e.g., butyrate) enhance energy supply by improving mitochondrial function [68], while tryptophan metabolites (e.g., kynurenine) modulate neuronal survival through AHR signaling [69]. The review also highlighted eight research hotspots for the next two decades, including the MGBA, FMT (fecal microbiome transplantation), gut microbiota, hypertension, TMAO, ischemic stroke, and neuroinflammation [2]. FMT is a therapeutic procedure involving the transfer of processed fecal material from a healthy donor into the GIT of a recipient to restore a balanced gut microbial eco-system. Recent investigations by researcher Dingzhi C. demonstrated that FMT administration in MCAO model rats resulted in (1) the restoration of intestinal homeostasis, (2) significant improvement in neurological function recovery, and (3) a marked reduction in cerebral infarct volume. Furthermore, the study revealed that FMT treatment effectively suppressed microglial hyperactivation in the ischemic brain tissue of these experimental animals [62].

### 4.1. Gut Microbiota-Derived Metabolites in Ischemic Stroke

#### 4.1.1. SCFAs Mitigate Neuroinflammation and Post-Stroke Recovery

**Figure 4:**
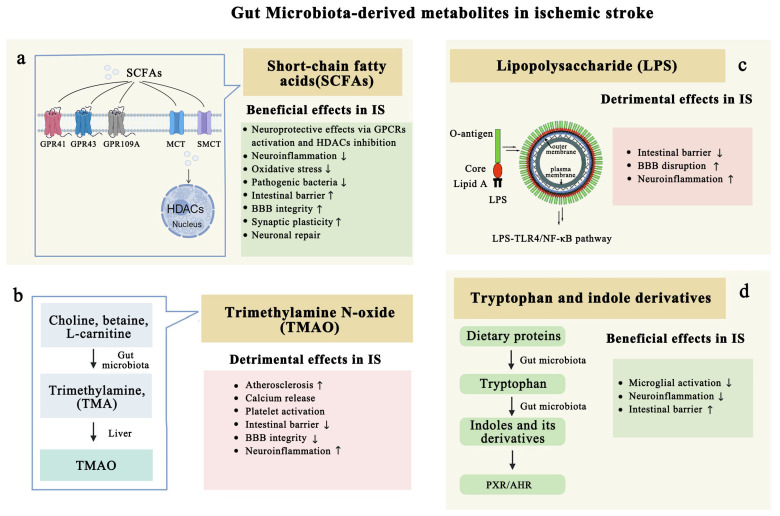
Gut microbiota-derived metabolites in ischemic stroke. (**a**) SCFAs exert their neuroprotective effects by acting as endogenous ligands for GPCRs and modulating gene expression by HDACs. (**b**) Trimethylamine N-oxide promotes neuroinflammation by initiating atherosclerosis, as well as through calcium overload, platelet aggregation, and an increase in BBB permeability. (**c**) Lipopolysaccharide (LPS) identifies TLR4, increasing NF-κB activation and neuroinflammation, and thereby disrupting the intestinal barrier and the BBB. (**d**) Tryptophan and indole derivatives activate microglial AHR and Progesterone X receptor (PXR) signaling to increase the intestinal barrier and inhibit microglial activation and neuroinflammation.

Building upon the role of gut microbiota in regulating inflammation, SCFAs have emerged as key microbial metabolites with neuroprotective potential. SCFAs are saturated fatty acids containing one to six carbon atoms, with acetate (C2), propionate (C3), and butyrate (C4) being the primary types present in the human body [70]. Numerous studies have demonstrated a link between SCFAs and various physiological processes in the human body, including the regulation of immunity [71,72], the maintenance of intestinal homeostasis [73,74,75], cholesterol metabolism [76], and the control of glucose and energy homeostasis [77,78,79]. In addition, SCFAs can influence brain function by directly crossing the BBB, where they modulate neuroinflammation and contribute to post-stroke recovery. SCFAs act as endogenous ligands for GPCRs (G protein-coupled receptors) and modulate gene expression by HDACs (inhibiting histone deacetylases) [80]. GPCRs are crucial for the nervous system’s precise response to external stimuli and internal states. SCFAs bind to GPCRs and modulate gene expression by HDACs, which are involved in chromatin remodeling and gene regulation, contributing to the regulation of inflammatory responses, neuronal survival, and tissue repair in the brain, highlighting their potential as therapeutic agents in stroke recovery [81] (Figure 4a). Examples include GPR43 (also known as FFAR2), GPR41 (FFAR3), and GPR109A, which are activated by butyrate and β-D-hydroxybutyrate. These receptors play a pivotal role in regulating the inflammatory response in the brain [80]. It has been demonstrated that FFAR2-deficient SPF (specific pathogen-free) mice displayed microglial defects similar to those observed in GF (germ-free) mice, indicating the importance of SCFA-receptor signaling in microglial function [82]. Additionally, one of the major SCFAs, acetate, demonstrated anti-inflammatory effects in Aβ-induced BV-2 microglial cells by upregulating GPR41 and inhibiting the ERK/JNK/NF-κB signaling pathway [42].

Acetate and butyrate have also been found to suppress inflammatory response in LPS-stimulated primary microglia through inhibiting histone deacetylase activity and NF-κB activation [83]. Furthermore, both propionate and butyrate reduce microglial activation and pro-inflammatory factors by inhibiting HDAC1 expression in GF mice, further emphasizing the regulatory role of SCFAs in neuroinflammation [84].

Another special kind of SCFA, the NaB (Sodium butyrate), a histone deacetylase inhibitor produced by the bacterium Bacteroides butyratrophicus, is able to cross the BBB. NaB has been shown to reduce oxidative stress within the brain, which in turn enhances the neuroprotectant insulin-like growth factor 1 (IGF-1) in peripheral tissues. The reduction in oxidative stress and IGF-1 expression ultimately leads to a decrease in the expression of pro-inflammatory cytokines in the serum. Consequently, NaB can effectively attenuate brain damage that may occur following a stroke [85].

#### 4.1.2. Trimethylamine N-Oxide Promotes Neuroinflammation and Worsens Stroke Outcome

TMAO, a metabolite produced by the gut, has been associated with stroke and various cardiovascular disorders, including atrial fibrillation, diabetes, congestive heart failure, chronic kidney disease, and both coronary and peripheral artery diseases [86]. It is widely acknowledged that TMAO is produced when dietary choline, phosphatidylcholine, and L-carnitine are metabolized by gut microbiota into trimethylamine (TMA), and subsequently absorbed and oxidized in the liver to form TMAO [87,88].

TMAO supplementation has been shown to impair cognition in mice by promoting neuroinflammation and disrupting the integrity of the intestinal barrier and the BBB, contributing to worsened stroke outcomes [89]. Notably, the probiotic Lactobacillus plantarum has been found to effectively reshape the gut microbiota and reduce TMAO levels in mice, thereby alleviating neuroinflammation and neurodegeneration [90] (Figure 4b). This suggests that modulation of the gut microbiome to reduce TMAO production could represent a promising strategy for mitigating neuroinflammation and improving stroke outcomes.

Clinically, elevated TMAO levels demonstrate three cardinal pathological effects: a pro-thrombotic state (increased platelet aggregation and thrombus formation), vascular endothelial impairment (decreased vasodilation capacity), and systemic inflammation (via NLRP3-dependent cytokine production) [86,91]. A nested case–control study within a Chinese ethnic group revealed a significant association between higher levels of TMAO and an increased risk of first-time ischemic stroke, particularly among hypertensive patients [92]. In terms of hypertension, TMAO drives a self-perpetuating pathological cycle linking hypertension and ischemic stroke [93]. First, TMAO promotes atherogenesis by upregulating scavenger receptors CD36 and SR-A1 in macrophages, thereby enhancing oxidized LDL uptake and accelerating atherosclerotic plaque formation. Second, TMAO directly increases stroke risk by inducing platelet hyperreactivity through enhanced calcium signaling and P-selectin expression, facilitating thrombus formation. Conversely, ischemic stroke exacerbates hypertension through neurohumoral mechanisms: damaged brain tissue releases angiotensinogen, which is converted to angiotensin II in circumventricular organs, leading to sympathetic overactivation and neurogenic hypertension [94].

Additionally, a cross-sectional case–control study found that TMAO levels were elevated in patients with ischemic stroke compared to healthy controls. Interestingly, lower levels of TMAO were observed in patients with mild stroke versus those with moderate and severe cases, as classified by the National Institutes of Health Stroke Scale. These findings suggest that elevated plasma TMAO levels at the time of admission may predict stroke severity, highlighting the potential role of TMAO as a biomarker in ischemic stroke prognosis [95].

#### 4.1.3. Lipopolysaccharide Disrupting the Intestinal Barrier and the Blood–Brain Barrier via Neuroinflammation

LPS are complex molecules embedded in the outer membrane of the cell wall in Gram-negative bacteria, consisting of three main components: lipid A, the core polysaccharide, and the O antigen. Serving as a protective barrier, LPS defends bacteria against external threats, including antibiotics, antibodies, and the complement system, thereby maintaining the stability and integrity of the cell wall. During the acute phase of an ischemic stroke, gut dysbiosis can lead to an overgrowth of opportunistic pathogenic Gram-negative bacteria, such as Escherichia coli. This can result in increased intestinal permeability, which, in turn, leads to ischemic brain injury via LPS-induced systemic inflammation [66].

As a primary component of LPS, lipid A is recognized as pathogen-associated molecular pattern (PAMP), acting as a potent inflammatory trigger for the host’s innate immune response through binding to TLR4. Upon entering the bloodstream, LPS can bind to LPS-binding protein (LBP) and is transferred to the CD14 present on the surface of monocytes and macrophages. This interaction triggers signaling cascades that lead to the production of pro-inflammatory cytokines, such as TNF-α, IL-1β, IL-6, and IL-12, further eliciting an inflammatory response.

In the context of ischemic stroke, LPS can induce vascular endothelial dysfunction by disrupting NO-mediated vasodilation through the inhibition of endothelial nitric oxide synthase (eNOS). It also promotes vascular inflammation via the activation of the MAPK/NF-κB pathway. These downstream signaling cascades contribute to atherosclerosis through the generation of oxygen free radicals, inflammatory cytokines, chemokines, and adhesion molecules. Furthermore, LPS is recognized as an endotoxin due to its ability to induce fever, shock, and other manifestations of systemic inflammatory response syndrome (SIRS). The inflammation and oxidative stress triggered by LPS can lead to endothelial cell apoptosis, further compromising the integrity of the vascular and BBB (Figure 4c). This disruption leads to increased BBB permeability, allowing the infiltration of inflammatory cells and contributing to the post-stroke brain tissue edema, emphasizing the crucial role of LPS-mediated neuroinflammation in stroke pathophysiology [96].

In a cohort study, researchers found that levels of plasma LPS, LBP, and soluble CD14 significantly increased from day 1 to day 6 after stroke when compared to control subjects. This elevation in LPS activity was correlated with a worse short-term outcome following the stroke [97]. In a separate study on diabetic mice, the oral administration of polymyxin B, a non-absorbable antibiotic, was shown to modify the gut microbiota, reduce plasma LPS levels, and thus improve metabolic endotoxemia. This treatment was also associated with enhanced post-stroke recovery and a reduction in neuroinflammation within the ischemic brain tissue [98]. To address these secondary injuries and their contributions to stroke pathology, interventions can be targeted at the underlying mechanisms. This includes the administration of TLR4 antagonists to block the LPS-TLR4 interaction, antioxidants to combat oxidative stress, or anti-inflammatory agents to reduce the inflammatory response. Such therapeutic interventions may play a crucial role in attenuating the inflammatory cascade and minimizing endothelial damage, which are critical steps in the recovery process following a stroke. By targeting these pathways, there is potential to improve clinical outcomes and reduce the severity of neurological deficits in stroke patients.

#### 4.1.4. Tryptophan and Indole Derivatives Modulate Neuroinflammation

Amino acids are essential in the production of bioactive molecules such as neurotransmitters, and their metabolism is influenced by the gut microbiota. Among these amino acids, tryptophan stands out as an essential amino acid that must be obtained through the diet. It serves as a biosynthetic precursor for a variety of microbial and host metabolites, including indole and its derivatives [99,100,101,102]. Tryptophan metabolism involving certain gut microbiota can lead to alterations in immune cell functions within the gut. This process is part of the complex interplay between the gut microbiota and the host’s immune system (Figure 4d).

There is a growing body of evidence suggesting that changes in the composition of the gut microbiota can significantly impact the MGBA by modulating tryptophan metabolism [103]. Population-based studies and nested case–control studies have highlighted the intricate relationship between tryptophan metabolism and both cardiovascular and cerebrovascular diseases. An inverse correlation between tryptophan levels and cardiovascular disease has been observed, along with a positive correlation between its metabolites, including kynurenine and serotonin, suggesting that the balance of tryptophan metabolism may be a critical factor in maintaining cardiovascular and cerebrovascular health [104]. A study by Liu et al. further supports the notion that tryptophan exerts a protective role in ischemic stroke. Their findings indicate that tryptophan or its metabolites could potentially serve as biomarkers or targets for therapeutic intervention in cerebrovascular disease [105]. Kan Gao and colleagues have shown that tryptophan metabolism, which produces serotonin, kynurenines, tryptamine, and indole compounds, is an important metabolic pathway in regulating the MGBA, providing further evidence of its critical role in stroke pathophysiology [106].

As one of the signaling molecules that regulate inflammatory and autoimmune responses, Indole and its derivatives are produced by the gut microbiota through the breakdown of tryptophan. These metabolites interact with the AHR, which is involved in both pro-inflammatory and anti-inflammatory pathways. The AHR’s role in cellular activities means that it also plays a significant role in neurological and neuropsychiatric conditions [107].

Microbiome-derived AHR ligands, such as IAA (indoleacetic acid), have been shown to exert anti-inflammatory effects, particularly within the CNS (Figure 4d). The study by Honarpisheh et al. suggests that pharmacological inhibition of the AHR after stroke can mitigate the harmful effects of kynurenine-mediated AHR activation and promote recovery. Furthermore, IAA’s ability to regulate microglia-mediated neuroinflammation points to its potential as a therapeutic strategy for enhancing neural repair and reducing inflammation after cerebral ischemia [108]. These findings underscore the importance of understanding the gut microbiota’s role in tryptophan metabolism and its impact on brain health. Future research may focus on developing treatments that target these metabolic pathways to improve outcomes in stroke and other neurological disorders.

### 4.2. Gut Microbiota-Related Neurotransmitters in Ischemic Stroke

#### 4.2.1. Serotonin: Inflammation and Protection

Serotonin (5-hydroxytryptamine, 5-HT) functions as a crucial neurotransmitter that plays a significant role in an array of physiological processes. It is intricately linked to numerous clinical disorders, including but not limited to migraines, depression, cardiovascular diseases, schizophrenia, and Alzheimer’s disease [109,110].

The GIT is a major site of serotonin production, with approximately 90% of the body’s serotonin being synthesized by enterochromaffin cells in the gut [111]. Given the role of gut microbiota in regulating this synthesis, there exists a complex interplay between the microbiome and serotonin metabolism.

**Figure 5:**
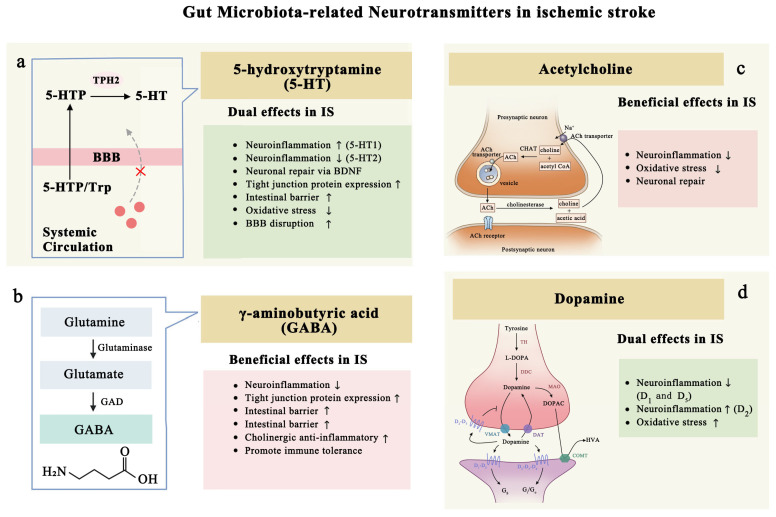
Gut microbiota-related neurotransmitters in ischemic stroke. (**a**) Serotonin (5-HT) engages with various 5-HT receptors on immune cells, eliciting pro-inflammatory effects (5-HT2 receptor) and anti-inflammatory responses (5-HT1 receptors and 5-HT4 receptors). (**b**) GABA, converted from glutamate, curbs immune activity, fosters immune tolerance and tight junction expression, and initiates cholinergic defenses, conferring anti-inflammatory and neuroprotective functions. (**c**) Acetylcholine binds to the α7-nAChRs on neural immune cells (microglia and astrocytes) to suppress the secretion of pro-inflammatory cytokines (IL-1β, IL-6, and TNF-α) and fosters neural regeneration, reducing oxidative stress. (**d**) Dopamine yields dual effects in post-stroke brain damage and neural recovery. The dopamine metabolite DOPAC triggers oxidative stress, intensifying neuroinflammatory responses. Dopamine modulates neuroimmune inflammation through engaging various dopamine receptors, offering both neuroprotective (DRD-1, DRD-3) and neuro-damaging (DRD-5) functions.

In addition to its role as a neurotransmitter, serotonin also presents in various immune cells, including T cells, macrophages, mast cells, dendritic cells, and platelets, indicating that serotonin has both neural and immunological functions [109]. While serotonin produced in the gut does not typically cross the BBB, certain precursors and derivatives can. These molecules can influence mood, sleep patterns, and neural processes once they reach the brain [110]. For example, 5-Hydroxytryptophan (5-HTP), an amino acid and direct precursor to serotonin, can cross the BBB, where it is converted into serotonin. In addition, N-acetyl serotonin, a serotonin derivative, can also pass into the brain and is involved in the synthesis of melatonin, the hormone that regulates sleep–wake cycles [112]. Melatonin itself can cross the BBB and is associated with various neural processes, including circadian rhythm regulation and sleep patterns (Figure 5a). Also, serotonin and its precursors can act as pro-inflammatory mediators, activating the immune response to various stimuli. This activation can lead to the production of pro-inflammatory cytokines, such as TNF-α, IL-13, and IL-6, which are involved in the inflammatory response, exerting both protective and detrimental effects according to their concentrations and the context in which they are produced [110]. This dual role of serotonin in inflammation underscores its complex involvement in neuroimmune interactions, particularly in the context of ischemic stroke and neuroinflammation [109].

In addition to these effects, serotonin also plays a pivotal role in stimulating the secretion of mucus through 5-HT receptors located on goblet cells, thereby further contributing to maintaining gut health [113]. The modulation of immune responses by serotonin signifies a complex neuroimmune interplay that is evident in gut inflammation, where both pro-inflammatory and anti-inflammatory consequences are observed.

Triptans, which are 5-HT1b/1d receptor agonists, are commonly used to treat migraines by inducing the vasoconstriction of meningeal blood vessels, inhibiting neuropeptide release, and providing rapid relief from headaches. These medications can significantly elevate the level of 5-HT in the serum [114]. Although effective in migraine relief, this may have implications for cerebrovascular health. A Danish case-crossover study suggests that the use of Treprostinil is associated with an increased risk of ischemic stroke and myocardial infarction. However, this risk is relatively low for individuals with a low underlying cardiovascular risk [115]. This indicates that 5-HT may be closely linked to cerebrovascular events, in a context-dependent manner.

Thomas C. Fung and colleagues demonstrated a significant correlation between the gut microbiota, particularly Turicibacter species, and 5-HT levels in mice. Specifically, the relative abundance of Turicibacter was positively associated with fecal and colonic 5-HT levels in both SPF and GF mice, as well as after inoculation with spore-forming bacteria of mouse or human origin. Furthermore, this research found that T. sanguinis can absorb 5-HT, and this process is inhibited by the SSRI (selective serotonin reuptake inhibitor) fluoxetine, suggesting that gut bacteria may influence host physiology and behavior by modulating 5-HT levels [113].

The serotonin reuptake transporter, also known as the 5-HTT (5-HT transporter), is encoded by the SLC6A4 gene, which regulates 5-HT content by transporting 5-HT from the synaptic cleft back to the presynaptic neuron [116]. Hypermethylation of the promoter region of the SLC6A4 gene reduces 5-HTT transport, leading to decreased 5-HT availability and impaired regulation of the 5-HTergic axis. This, in turn, promotes platelet aggregation and local vasoconstriction, increasing the risk of composite cardiovascular and cerebrovascular events [117].

Several studies have shown that SLC6A4 gene methylation is associated with functional outcome and rehabilitation following stroke [118,119]. Kang et al. found that high methylation levels in the promoter region of the SLC6A4 gene were associated with an increased risk of composite cardiovascular events, including recurrent stroke, myocardial infarction, and vascular death, up to 14 years after ischemic stroke [120]. The 5-HTergic system interacts with the HPA axis, affecting the release of inflammatory factors and immune mediators in the body, which may hinder stroke recovery [121]. In conclusion, during methylation, the SLC6A4 gene, which influences the 5-HTergic system, has a profound effect on hippocampal and motor neuron plasticity. It also participates in the balance of the HPA and KYN axes and influences the release of inflammatory factors and immune mediators, thereby affecting the long-term prognosis of stroke.

#### 4.2.2. GABA: Post-Stroke Neurofunctional Recovery

As the major inhibitory neurotransmitter in the CNS, GABA is involved in a wide range of physiological processes, including motor control, anxiety regulation, and sleep. Both in vivo and in vitro studies indicate that GABA inhibits glutamate-mediated excitotoxicity and enhances functional recovery after ischemic stroke by modulating long-term potentiation in damaged neurons. This underscores the crucial role of GABAergic neurotransmission in stroke recovery, which is directly linked to the abundance, type, and function of GABA receptors. These receptors are located on the surface of various immune cells, such as T cells, B cells, and macrophages, further implicating GABA in post-stroke immune modulation [122].

Studies have consistently shown that GABA inhibits glutamate-induced neurotoxicity and supports functional recovery after stroke (Figure 5b). Ying et al. discovered that GABA-A receptors in the dorsal striatum are essential for motor recovery following exercise. Exercise training may increase synaptic plasticity and GABA levels in MCAO mice by activating cortical–striatal circuits, thereby improving neurological outcomes [123]. Rhita et al. found that endozepines, ligands for the benzodiazepine site of GABA-A receptors, enhance the activity and excitability of neurons in the cerebral cortex. They proposed that post-stroke neurogenesis can be controlled by endozepines to optimize neurofunctional recovery [124]. However, a double-blind, randomized clinical trial of S44819, a GABA-A α5 antagonist, failed to enhance clinical outcomes in ischemic stroke patients, highlighting the complexity of GABAergic modulation in clinical settings [125].

The gut microbiome has emerged as a key player in the synthesis and regulation of GABA. Certain gut microbes, such as Bacteroides, Bifidobacterium, and Lactobacillus, are recognized as GABA producers, and alterations in the composition of the gut microbiota can lead to changes in GABA concentrations [126]. This suggests a potential correlation between the composition of the gut microbiota and the regulation of GABA levels. Janik et al. demonstrated that the probiotic Lactobacillus rhamnosus can enhance various CNS metabolites, including GABA, and influence GABA receptor expression [127]. Similarly, Camile Petitfils and colleagues proved that the production of lipopeptide GABA by commensal bacteria could be one of the mechanisms through which the gut microbiota communicates with the host, contributing to the maintenance of intestinal homeostasis [128].

Furthermore, changes in diet can modify gut microbiota, subsequently affecting GABAergic signaling. Olson et al. found that a ketogenic diet increased GABA and glutamate levels in the hippocampus, improved the GABA/glutamate ratio, altered the excitation/inhibition balance, and provided neuroprotective benefits. These effects were mediated by changes in gut bacteria, particularly Eckermannia and Actinobacillus [129]. Recent studies also found that GF mice exhibit low brain glutamine levels, while those administered probiotics showed a higher GABA/glutamate ratio [130].

Glutamate, mostly synthesized from the nonessential amino acid glutamine and α-ketoglutarate, serves as a biological precursor to GABA and glutamine. The two primary enzymes facilitating glutamate synthesis and metabolism are aspartate aminotransferase and glutamate dehydrogenase, while the synthesis of GABA is catalyzed by glutamate decarboxylase [131]. Increased glutamate levels can enhance GABA and glutamine synthesis, maintaining a balance between these neurotransmitters under normal conditions. However, during the onset of ischemic stroke, glutamate plays a major role in excitotoxicity. It is traditionally viewed as the first messenger to activate the N-methyl-D-aspartate receptor (NMDAR)-dependent cell death pathway, contributing to oxidative stress, inflammation, and other pathological mechanisms following cerebral ischemia. The first connection between glutamate and stroke damage was established in 1959 when Van Harreveld discovered that applying glutamate to rabbit brain tissue led to increasing depression [132].

During an acute stroke episode, glutamate levels quickly rise in the ischemic area of the brain [133]. In vitro, hypoxic neuronal death can be inhibited by tetrodotoxin, a voltage-gated sodium channel blocker that prevents action potentials, or by magnesium, which blocks NMDA receptors and synaptic glutamate release. Thus, several sodium channel blockers have been developed to inhibit ischemic glutamate release, which can effectively lower glutamate levels and reduce neuronal death following cerebral ischemia in vivo [134]. A recent study discovered that glutamate exacerbates brain damage after stroke by binding to acid-sensitive ion channels [135]. These studies demonstrate that inhibiting ischemic glutamate release can mitigate excitotoxicity caused by ischemic stroke during the initial stage.

#### 4.2.3. Acetylcholine: Anti-Inflammation and Neuronal Regeneration

ACh is a monoamine neurotransmitter synthesized by choline acetyltransferase and is widely distributed in the nervous system (Figure 5c). It plays a crucial role in various processes, including arousal, sleep, and consciousness. Borovikov’s team first introduced the concept of the cholinergic anti-inflammatory pathway [136], a neural-immune regulatory mechanism in which peripheral inflammatory signals are relayed to the NTS (nucleus tractus solitarius) via vagal afferent fibers. Within the NTS, these signals stimulate the DMN (dorsal motor nucleus of the vagus), initiating efferent vagus nerve activity. This efferent response induces β2AR (β2 adrenergic receptor) activation on T cells, prompting ACh release. ACh then binds to α7nAChRs (α7 nicotinic ACh receptors) on macrophages, suppressing NF-κB-mediated pro-inflammatory signaling and downregulating the production of key cytokines, including TNF-α, IL-1β, and IL-6. The vagal modulation of inflammation primarily occurs through these cholinergic anti-inflammatory pathways, which are more dynamically responsive to inflammation than the humoral immune response [137].

Experimental evidence has demonstrated the existence of cholinergic anti-inflammatory pathways in animal models of sepsis, endotoxemia, and myocardial ischemia [138]. For instance, studies have shown that non-invasive vagus nerve stimulation, applied before and after emergency PCI (percutaneous coronary intervention) in patients with AMI (acute myocardial infarction), significantly reduces inflammatory markers such as IL-1β and TNF-α in the blood. This approach has been associated with a decrease in reperfusion arrhythmias, the mitigation of myocardial damage, and an enhancement of cardiac function. These positive outcomes in AMI suggest that similar benefits could be extended to AIS, given the shared inflammatory and ischemic mechanisms between myocardial infarction and stroke [139].

The cholinergic system plays a pivotal role in modulating the responses of astrocytes and microglia to brain injury. The anti-inflammatory pathway in both microglia and astrocytes is mediated by α7nAChR. The binding of Ach to α7nAChR on microglia leads to the downregulation of pro-inflammatory cytokines [140,141]. In MCAO mice, the stimulation of α7nAChRs with their agonist choline resulted in an increased expression of α7nAChRs, as well as a reduction in infarct volume and neurological deficits, supporting the therapeutic potential of cholinergic modulation in promoting post-stroke neurofunctional recovery [142].

In cerebral ischemic models, AChE (acetylcholinesterase) inhibitors have been shown to protect against neuronal death by activating nicotinic Ach receptors. A Swedish cohort study including 44,288 dementia patients indicated that the use of AChE inhibitors may be associated with a lower risk of ischemic stroke and mortality [143]. This underscores the potential of AChEIs as a therapeutic strategy for preventing ischemic stroke.

VNS (vagus nerve stimulation) is another promising neuroprotective intervention with demonstrated efficacy in mitigating cerebral ischemia–reperfusion injury. It exerts neuroprotective effects by regulating the redox state through the activity of miR-210, a microRNA involved in cellular stress responses [144]. Emerging evidence suggests that VNS, during tactile rehabilitation, can promote somatosensory recovery [145].

The administration of nicotine has been shown to downregulate the expression of IL-1β, IL-6, and TNFα in astrocytes [141] via the cholinergic anti-inflammatory pathway. Schuhmann et al. discovered that midbrain electrical stimulation could activate the cholinergic anti-inflammatory pathway, increasing Ach levels in the brain, which in turn enhances the activities of acetylcholinesterase and choline acetyltransferase, resulting in a decrease in the expression of pro-inflammatory cytokines such as IL-1β and TNF-α and an increase in the expression of anti-inflammatory cytokines such as IL-10 [146]. These findings highlight the importance of the cholinergic system in neuroinflammation and suggest potential therapeutic targets for post-stroke brain recovery.

#### 4.2.4. Dopamine (DA): Dual Roles After Ischemic Stroke

DA, a key catecholamine neurotransmitter, is integral in regulating immune inflammation. Under normal physiological conditions, DA exerts an inhibitory effect on neuroinflammation. However, under pathological conditions, dopaminergic neurons may produce heightened levels of ROS, initiating an inflammatory response. This uncontrolled inflammation can lead to a vicious cycle associated with neurodegenerative diseases, where ongoing damage and inflammation exacerbate neuronal loss and dysfunction (Figure 5d).

DA is also crucial in preventing the activation of NLRP3 inflammasomes and the subsequent inflammatory responses they trigger, largely through the engagement of DRD1 (DA receptor D1) signaling pathways. Furthermore, DA produced via vagus nerve electrical stimulation has a broad and significant suppressive impact on various inflammatory factors, including TNF-α, MCP-1, IL-6, and IFN-γ. This anti-inflammatory effect is primarily mediated through the activation of D1-like receptors, which are key in modulating inflammatory responses. In a study led by Yang Shuo, it was demonstrated that DA inhibits macrophage NF-κB inflammatory responses mediated by the TLR2 (Toll-like receptor 2) pathway via DRD5 (DA receptor 5) [147]. These findings underscore the complex role of DA in immune regulation and suggest that targeting dopaminergic pathways could be a promising strategy for treating inflammatory conditions and, potentially, neurodegenerative diseases.

DA agonists have been widely explored in clinical settings for their potential to enhance post-stroke recovery. DA agonists stimulate central dopamine receptors, increasing DA release and modulating its effects on neuronal function [148]. Clinical trials have obtained variable results regarding the effects of DA agonists in post-stroke treatment.

For example, voriconazole and riturabine have shown positive efficacy in restoring the level of consciousness and neurological function in comatose patients following ischemic stroke [149]. In a randomized, double-blind, controlled trial conducted by Gorgoraptis et al., patients with lateral spatial neglect and left-sided motor deficits following a right hemisphere stroke were recruited. The results suggested that rotigotine, a DA agonist, may be effective in improving lateral spatial neglect [150]. However, the study also found that rotigotine failed to improve the recovery of motor control, which further proved by Dr. Gary. In a similar randomized, double-blind, controlled clinical trial conducted by Dr. Gary and his team, it was found that the addition of compounded carbidopa to conventional pharmacological and physiological therapy did not appear to improve motor function after a stroke [151]. Further research is needed to identify the complex and context-dependent effects of dopamine agonists in post-stroke rehabilitation.

## 5. Points of Intervention to Improve Microbiome–Gut–Brain Axis

The gut microbiota has been found to interact with the brain via the MGBA, regulating various physiological processes. Researchers have investigated the communication between gut bacteria and the brain, highlighting the importance of the balance between beneficial and harmful bacteria within the gut microbiota as a potential target for therapeutic interventions. This section outlines two potential points of intervention for regulating the MGBA: the intestinal barrier and the BBB.

### 5.1. Intestinal Barrier Restoration

The IEB (intestinal endothelial barrier) is essential for maintaining intestinal homeostasis, serving as a physical barrier and a coordinator of immune responses. It comprises epithelial cells, goblet cells, Paneth cells, and enterochromaffin cells. Communication between the intestinal microbiome and peripheral nerve cells within the gut–brain axis occurs through three interrelated pathways. Endothelial cells in the gut respond to signaling molecules released by gut bacteria by initiating the release of neuropeptides. The receptors on EECs (enteric epithelial cells) are specifically located on vagal sensory neurons that project into the gut, influencing various physiological functions associated with digestion and metabolic regulation [152,153,154,155].

Moreover, evidence suggests that an individual’s age can affect the integrity of the IEB following ischemic stroke. With aging, the gut barrier weakens, allowing pro-inflammatory substances from gut microbes and harmful bacteria to enter the bloodstream, leading to systemic inflammation and neuroinflammation [156]. In vivo studies in animal models have shown that stroke significantly disrupts intestinal homeostasis, with older mice exhibiting more pronounced effects compared to younger ones, suggesting that age may be a critical factor in the extent of post-stroke gut barrier disruption [157].

The current evidence suggests that stroke can lead to a disruption in the integrity of the IEB, resulting in the deterioration of the intestinal villous epithelium, increased permeability, and compromised tight junctions, which facilitates the leakage of harmful substances into the bloodstream, leading to a reduction in mucus production, further weakening the intestinal barrier and increasing susceptibility to enterogenic sepsis [158]. The majority of γδT cells in the human body are located on the surface of the intestinal epithelium, where they play a role in the innate immune response of the intestine. Following ischemic stroke, γδT cells migrate from the gut through the peripheral circulation to the brain membrane and secrete IL-17 into the damaged brain tissue, inducing the production of increased chemokines in the brain parenchyma. This eventually leads to a massive influx of neutrophils and exacerbates ischemic neuroinflammation [159].

Many gut microbes and their byproducts can influence the outcome of a stroke by modulating the intestinal mucosal barrier. A high fiber intake has been shown to promote the growth of fiber metabolizers and SCFA producers, which can enhance mucus secretion and maintain a healthy mucus layer [160,161]. On the other hand, low-fiber diets in mice led to an increase in the population of mucus-degrading bacteria, with a thinner mucus layer and an elevated risk of infection [162]. Other metabolites involved in maintaining intestinal barrier integrity include indole and its derivatives. Compounds such as indole-3-ethanol, indole-3-pyruvate, and indole-3-aldehyde reinforce the apical junctional complex, thereby enhancing the resilience of the intestinal barrier [163]. Furthermore, these indoles can inhibit microglial and NLRP3 inflammasome activation by engaging the microglial AHR, mitigating post-stroke neuroinflammation [164].

### 5.2. Blood–Brain Barrier Restoration

The BBB is a sophisticated physiological and biochemical barrier that maintains brain homeostasis by regulating the exchange of substances between the brain and its external environment. It is responsible for removing harmful metabolic byproducts and internal endotoxins from the brain [165]. The integrity of the BBB is maintained by tightly joined, non-porous endothelial cells connected by tight and adherent junctions. These endothelial cells share a basement membrane with pericytes and astrocytes, along with neurons and microglia, forming neurovascular units that are crucial for maintaining BBB integrity. Inflammatory molecules such as IL-6 and IL-8 are believed to disrupt neurovascular function and may contribute to the increased vascular permeability observed in the BBB during inflammation [166,167]. These disruptions facilitate the infiltration of peripheral immune cells and inflammatory mediators into the CNS, exacerbating neuroinflammation and contributing to the pathogenesis of neurological disorders.

Numerous studies suggest that metabolites produced by the gut microbiota significantly influence the regulation of BBB integrity [168,169,170,171,172]. SCFAs, among the most-studied microbial metabolites, have a notable impact on BBB integrity. Research using in vitro BBB models has revealed a protective effect of SCFAs on maintaining the barrier.

The role of TMAO in BBB function is more variable. Some studies link TMAO to neuroinflammation due to its ability to activate microglia and astrocytes. TMAO has also been associated with negative impacts on neuronal health, including neuronal aging, oxidative stress, and alterations in synaptic plasticity [89,173,174,175,176,177,178,179]. These findings suggest that elevated TMAO levels may exacerbate the inflammatory processes underlying neurodegenerative diseases. However, it has also been found that the chronic administration of low doses of TMAO can protect against LPS-induced BBB damage and memory deficits in C57Bl/6J mice [168]. This raises the possibility that TMAO’s effects on the BBB may depend on dose and the specific context of its administration.

Inflammation is known to have an adverse impact on BBB function [167]. Pro-inflammatory substances have the potential to compromise the integrity of the BBB, which could allow substances from the bloodstream to enter the brain. This breach in the BBB could establish a connection between the CNS and the peripheral immune system, potentially leading to neuroinflammatory conditions.

## 6. Stroke Treatment Based on Microbiota–Gut–Brain Axis

It is well-established that a marked reduction in gut microbiota species diversity is a hallmark of microbiota dysbiosis following ischemic stroke [66]. Tan et al. demonstrated that AIS induces gut dysbiosis, which in turn affects the neuroinflammatory response and exacerbates stroke outcomes [180]. Furthermore, gut dysbiosis can cause an imbalance in T cell subsets, either worsening or mitigating ischemic brain injury. In line with this, a cohort study comparing the gut microbiota of 124 stroke patients discovered a significant enrichment of Enterobacteriaceae in those with poor recovery compared to those who recovered well. In an experimental study, antimicrobial treatment was administered to MCAO mice, confirming the effectiveness of inhibiting the overgrowth of Enterobacteriaceae in the context of stroke [66].

Benakis C. et al. discovered that alterations in the gut microbiota induced by antibiotics can diminish brain damage after stroke in mice. They also suggested that this protective effect could be transferred via fecal microbiota transplantation [48]. Considering the increasing evidence highlighting the potential of gut microbiota manipulation as a therapeutic target for AIS, both experimental and clinical studies have been conducted to explore this approach.

### 6.1. Diet and Stroke

Diet, as a modifiable factor, exerts an influence on the gut microbiota across both short- and long-term periods. Several studies proved that vegetable consumption may be associated with a reduced risk of stroke [181,182,183]. A large European cohort study revealed that a higher intake of fruits, vegetables, fiber, and dairy products was linked to a lower risk of ischemic stroke, while a higher consumption of red meat was associated with an increased risk. Additionally, higher egg intake was found to correlate with an elevated risk of hemorrhagic stroke [181]. Similarly, a Taiwanese case–control study found that adhering to a vegetarian diet in Taiwan was associated with a lower risk of both ischemic and hemorrhagic stroke [182].

In 2023, a case–control study among the Lebanese population investigated the impact of the DASH (Dietary Approaches to Stop Hypertension) diet on ischemic stroke and found that the diet was protective against such strokes and associated with less disability [184]. The DASH diet emphasizes the consumption of vegetables, fruits, whole grains, low-fat dairy, lean meats, and nuts, along with a significant reduction in sodium intake [185]. In contrast, a recent study indicated that a plant-based diet might actually increase the risk of stroke compared to red meat consumption, suggesting that an exclusively plant-based diet may not always confer the expected health benefits [186].

The ketogenic diet, which is very high in fat and virtually devoid of carbohydrates, has yielded conflicting results regarding its impact on gut microbiota. While some research indicates that such diets may disrupt the balance of the gut bacteria and lead to inflammation [187,188], others have highlighted the potential benefits of ketogenic diets in the context of stroke-related diseases, [189,190] as diet-induced ketosis may have a positive relation with the development of an anti-inflammatory microglial phenotype [189].

The relationship between coffee consumption and stroke risk remains contentious. A study [191] involving 13,462 stroke cases and 13,488 controls from the INTERSTROKE database found that high coffee intake was associated with an increased stroke risk, whereas tea consumption was linked to a reduced risk. In contrast, a cohort study using UK Biobank data [192] suggested that drinking coffee and tea, either separately or together, was associated with a lower risk of stroke and dementia. Another interesting study found that carbonated beverages were linked to higher odds of ischemic stroke and intracerebral hemorrhage, while high water intake correlated with lower ischemic stroke odds, with notable regional variations [193]. To date, only a fraction of research has explored the interplay between diet, stroke, and gut microbiota. A comprehensive investigation into dietary impacts on gut microbiota could offer novel insights in the prediction of stroke outcomes (Table 3).

In summary, dietary interventions targeting post-stroke neuroinflammation and the subsequent modulation of neurological outcomes through the MGBA still face several practical limitations. For instance, the precise dose–response relationship between dietary fiber intake and stroke incidence or severity remains unclear. Additionally, significant inter-individual variability—such as differences in baseline gut microbiota composition, prior antibiotic use, and long-term dietary patterns before stroke onset—may introduce bias into the observed outcomes.

### 6.2. Antibiotic and Probiotics or Prebiotics Therapy

Antibiotic treatment can significantly alter the composition and function of the intestinal microbiota, potentially disrupting the microbiome’s homeostasis and influencing stroke outcomes [194]. The gut microbiota plays a crucial role in maintaining immune regulation, metabolic balance, and neurological function. When antibiotics disturb this delicate ecosystem, dysbiosis can occur, leading to a reduction in beneficial bacteria (e.g., *Lactobacillus* and *Bifidobacterium*) and an overgrowth of pathogenic or opportunistic microbes [195]. This imbalance may compromise gut barrier integrity, increase systemic inflammation, and modulate immune responses, all of which can impact stroke pathogenesis and recovery [196]. Interestingly, while antibiotic-induced microbiota disruption is generally detrimental, some studies suggest that the targeted modulation of gut bacteria may have therapeutic potential in stroke. Utilizing antibiotics to target gut microbiota can lead to the production of inflammatory factors such as IL-1, IL-6, and iNOS, which may reduce the formation of infarcted areas. As mentioned in Section 3.2 [48], Benakis C. et al. conducted animal experiments that demonstrated that gut microbial changes due to antibiotic treatment following ischemic stroke can result in modifications to dendritic cells. These alterations disrupt intestinal immune homeostasis, leading to an increase in regulatory T cells and a decrease in IL-17-producing T cells, mitigating ischemic brain damage in MCAO mice. Importantly, whether neuroprotection is mediated by gut microbiota is dependent on the balance between IL-10 and IL-17, suggesting an intricate connection between gut microbiota and the immune system. However, the use of broad-spectrum antibiotics, which reduce gut microbial populations, does not affect brain damage within the first day post-stroke, but may suppress systemic immunity, leading to increased mortality between days 5 and 7 following stroke [197]. These findings highlight that the role of antibiotics in preventing and treating stroke remains a topic of debate.

Probiotics are defined as “live microorganisms that, when administered in sufficient quantities, confer health benefits on the host.” While prebiotics are “substrates selectively utilized by host microorganisms to promote health”. From a microbiological perspective, prebiotics are non-digestible dietary compounds that selectively stimulate the growth and/or activity of beneficial gut microbiota, thereby conferring indirect health benefits. In contrast, probiotics are live microorganisms which, when administered in adequate amounts, directly confer a health benefit to the host by modulating gut microbial composition and function. Both probiotics and prebiotics are effective in restoring beneficial gut microbiota and metabolic functions, enhancing the production of SCFAs and thus improving the integrity of biological barriers and reducing systemic LPS levels. As a result, systemic inflammation and glial activation decrease, which mitigates neurodegenerative disease pathology.

Prebiotics, which are non-digestible compounds found in high-fiber foods like garlic, onions, bananas, asparagus, oats, and chicory root, exert beneficial effects on the host by influencing the composition and activity of the intestinal microbiota through microbial metabolism [198]. Commonly studied prebiotics include inulin, fructooligosaccharides (FOS), and galactooligosaccharides (GOS), which promote the growth of Lactobacillus and Bifidobacterium species [199,200,201,202,203].

The administration of probiotics, which are common in fermented foods like yogurt, kefir, sauerkraut, kimchi, miso, and supplements, has been shown to restore intestinal microbial balance, reduce the production of TMAO, increase SCFA production, alleviate damage to tight-junction proteins, and decrease both adaptive and innate immune activation, thus minimizing post-stroke inflammatory damage [204,205,206]. A higher risk of stroke has been associated with an imbalance in the gut microbiota, characterized by an increase in opportunistic pathogens and lactate-producing bacteria and a decrease in butyrate-producing bacteria [207]. A combination of early enteral nutrition and probiotics can effectively improve the nutritional status of stroke patients, regulate intestinal flora and mucosal barrier function, and enhance immune function, helping to reduce the incidence of infectious complications and gastrointestinal motility disorders. This therapeutic approach also contributes to faster recovery and better outcomes for stroke patients [208,209]. Additionally, probiotic treatment can protect gut barrier integrity and increase the expression of brain GLP-1 receptors and the secretion of gut GLP-1, a hormone that regulates 5-HT levels and appetite [15].

Despite their promising benefits, the use of probiotics to prevent and treat strokes caused by gut bacteria remains a conflict. Some probiotic strains may exacerbate inflammation by promoting M1 macrophage polarization [210,211]. A randomized controlled trial involving elderly care-home residents found that probiotics had no significant effect on plasma immune mediator levels, neutrophil and monocyte phagocytosis, or blood culture responses to immune stimulation [212]. This has raised concerns regarding the varying effectiveness of probiotics across different populations and conditions. Challenges in probiotics use include their susceptibility to a low gastric pH and digestive enzymes, which can lead to inactivation and reduced bioactivity. Moreover, biases can be introduced throughout the sample processing stages, including collection protocols, preservative selection, storage temperature, DNA extraction, library preparation, sequencing, and bioinformatics analysis [213] (Table 4).

### 6.3. Fecal Microbiota Transplantation

FMT has emerged as an innovative therapeutic approach, receiving attention due to its potential to modulate the gut microbiota and treat various conditions. FMT involves the transfer of stool from a healthy donor to a recipient, aiming to restore the recipient’s gut microbiota balance. This method is being explored as a treatment for neurological disorders, including stroke. To date, much of the research on FMT for stroke has been conducted in animal models. Studies have shown that FMT can exert neuroprotective effects by reducing pro-inflammatory bacteria and gut microbiota metabolites, as well as diminishing inflammatory responses and oxidative stress in the brain [214]. Research by Benakis et al. indicates that recolonizing mice with a dysbiotic microbiome can induce pro-inflammatory T-cell polarization in both the intestinal immune compartment and the ischemic brain. Additionally, therapeutic FMT has been shown to normalize brain lesion-induced dysbiosis and improve stroke outcomes in animal models [48]. Furthermore, the therapeutic transplantation of fecal microbiota has been demonstrated to normalize brain lesion-induced dysbiosis and improve stroke outcome [54]. Based on these findings, it is hypothesized that gut ecological dysregulation can significantly impact neuroinflammation, metabolism, and immune homeostasis following brain injury, suggesting that FMT could be a promising strategy for mitigating these effects in stroke patients. However, further research is needed to translate these preclinical findings into clinical practice and to address potential safety and efficacy concerns.

The gut microbiota exerts profound effects on human physiology by modulating host immunity, metabolic processes, and neurological functions through specialized bacterial communities. Of particular significance are the Lactobacillus species, Gram-positive, facultative anaerobic bacteria that dominate mucosal surfaces and the intestinal tract, where probiotic strains including L. acidophilus and L. rhamnosus strengthen epithelial barrier function, regulate immunological activity, and competitively exclude pathogenic microorganisms [215]. Equally noteworthy is Akkermansia muciniphila, a mucolytic Gram-negative anaerobe that maintains intestinal homeostasis through its unique capacity to utilize mucin as its primary energy source. Its colonization has been inversely correlated with systemic inflammation and metabolic syndrome, while it is positively associated with enhanced insulin signaling [216,217]. In contrast, Escherichia coli exemplifies the dichotomous nature of gut symbionts: commensal variants participate in essential physiological processes including vitamin K biosynthesis and niche occupation, whereas enteropathogenic serotypes such as O157:H7 possess virulence factors that can precipitate life-threatening gastroenteritis, illustrating the delicate balance between microbial mutualism and pathogenicity in the gastrointestinal ecosystem [218,219]. Laboratory studies have revealed that patients with ischemic stroke often exhibit a higher prevalence of opportunistic pathogens such as Bifidobacterium, Oscillibacter, and Enterobacter, and lower levels of beneficial genera like Faecalibacterium and Bacteroides in their feces or intestinal contents compared to healthy controls [220,221,222,223]. In a case–control study conducted by Na Li, 30 cerebral ischemic stroke (CI) patients and 30 healthy controls were enrolled to compare their fecal gut microbiota profiles using Illumina sequencing of the 16S rRNA gene. The result found that CI patients had significant dysbiosis, with an enrichment of short-chain fatty acid-producing bacteria such as Odoribacter and Akkermansia [224]. However, the reliability of these findings is limited due to the small sample size and the lack of consideration of other factors that could influence gut microbiota, such as dietary habits and medication use. These results collectively suggest that the composition of the gut microbiota may contribute to an individual’s risk of stroke.

The long-term outcomes of FMT as a therapeutic intervention are not yet fully understood, and some conflicting results have been reported. For instance, Vendrik et al. observed an increase in mortality following FMT in MCAO mice models [225], questioning the safety of this therapy. Regarding the application of FMT in stroke, only a limited number of human studies have been completed or are ongoing. To clarify the role of FMT in the context of stroke, substantial, double-blind, randomized controlled trials will be necessary.

## 7. Conclusions

The escalating global incidence of AIS, coupled with the limited range of treatment options, underscores the urgent need for the development of innovative therapeutic strategies. Disruption of the gut microbiome’s equilibrium negatively affects glial cells, compromising the integrity of both the intestinal barrier and the BBB. Metabolites resulting from gut dysbiosis, such as TMAO, SCFAs, and 5-HT, have been linked to the exacerbation of cerebrovascular damage and the progression of disease. Dietary choices have the potential to modulate these metabolites, indicating that dietary modifications could either increase or reduce the risk of stroke. Preclinical studies support the use of probiotics, prebiotics, and FMT as potential interventions to dampen glial overactivation and improve neurological function, thereby restoring the structural integrity of the intestinal and BBB barriers.

Nevertheless, current animal models are inadequate in fully capturing the complex dynamics of the human microbiome and its associated pathobiological mechanisms. The translation of laboratory findings into clinical practice remains a challenge, emphasizing the critical need for ongoing research efforts to decipher the mysterious nature of the MGBA and to leverage its full therapeutic potential. Conditions that predispose individuals to stroke and its aftermath may indeed position the gut microbiota as a novel and promising target for therapeutic intervention.

## Figures and Tables

**Table 1:** Post-stroke Inflammatory cells in MGBA.

Cell Types	Mechanisms	
Microglia	Pro-inflammation	M1 phenotype →Secretion of pro-inflammatory TNF-α, IL-1β, IL-6, IL-12, and IL-23. Ischemic core-associated microglia (ICAM) summoning peripheral immune cells to further infiltrate the CNS.
	Anti-inflammation	M2 phenotype → Secretion of anti-inflammatory IL-10. Ischemic penumbra-associated microglia (IPAM) boost anti-inflammatory metabolic properties and neuro-/myelin-protection.
Astrocyte	Pro-inflammation	TLR4/NF-κB: Induces release of IL-1β and TNF-α, amplifying neuroinflammation. C3a-C3aR: Complement activation promotes CSPG deposition/ fibrosis→ Impedes axonal regeneration.
	Anti-inflammation	Glutamate Uptake: Reduces excitotoxicity. BDNF Secretion: Maintains synaptic plasticity Astrocyte fibrosis: a physical barrier to limit inflammatory spread.
Dendritic cell	Pro-inflammation	NF-κB activation → Upregulates IL-6, TNF-α, IL-1β. DCs in peripheral lymph nodes upregulate CD40/CD80 → Activate naïve T cells (Th1/Th17) → T cells migrate to the brain via disrupted BBB:(1) Th1 cells: release IFN-γ → Drives microglial M1 polarization; (2) Th17 cells: release IL-17A → Activates astrocytes → CXCL1 production → Neutrophil recruitment.
	Anti-inflammation	DCreg surface PD-L1 binds to PD-1 on T cells → Inhibits TCR signaling → Suppresses IFN-γ (Th1) and IL-17 (Th17) production, Reducing T cell-mediated neuroinflammation and neuronal damage DCreg overexpress indoleamine 2,3-dioxygenase (IDO): (1) Depletes local tryptophan (Trp) → Blocks T cell proliferation (G1 phase arrest); (2) accumulates kynurenine (Kyn) binds AHR on T cells → Induces apoptosis/anergy.
Other immune components	Pro-inflammation	Th1 Cells: IFN-γ binds to microglial IFN-γR → Activates STAT1/JAK2 pathway → Drives M1 polarization. Th17 Cells: IL-17A binds to astrocytic IL-17R → NF-κB → CXCL1/CXCL2 release → Neutrophil chemotaxis.
	Anti-inflammation	Regulatory T Cells activate STAT3-dependent IL-10 transcription → Binds to IL-10R on microglia → Suppresses NF-κB signaling: Downregulates pro-inflammatory cytokines (TNF-α, IL-1β). attenuates microglial M1 polarization and oxidative stress

**Table 2:** The Key Differences Between Reginal Heterogeneity in Ischemic Brain.

Parameter		Core Infarction	Penumbra Region	References
Pathology		Irreversible	Reversible	[46]
Neuroimaging	MR	1. DWI: Hyperintense signal (ADC < 620 × 10−^6^ mm^2^/s) 2. FLAIR: hyperintense > 24 h	1. PWI-DWI mismatch: a mismatch volume on PWI and DWI lesion (PWI-Tmax > 6 s) 2. FLAIR: Negative	[22] [23]
	CT	1. CBV < 40%; CBF < 30% 2. Tmax: No flow (unmeasurable)	1. CBF-CBV mismatch (CBF < 30%; CBV normal/mildly↑) 2. Tmax > 6 s	[24]
Neuro- inflammatory Biomarkers	ROS	Severe ↑ (Nox2-driven, persistent)	Moderate↑ (transient, Nrf2-inducible)	[45,46] [55]
	Cytokines	TNF-α↑↑ IL-1β↑↑ (dominant cytokines)	IL-10↑, TGF-β↑ (mixed kinds of both destructive and protective cytokines)	[30]
	Microglia	M1-polarized (pro-inflammatory)	M2-polarized (anti-inflammatory)	[30,31]
	Cell Death	Necroptosis	Apoptosis	[46]
Clinical Implications		High risk with reperfusion therapy (hemorrhagic transformation)	Primary target for reperfusion therapy (thrombectomy/thrombolysis)	[22,23,24,43]
Outcomes		Functional Loss	Neuro/angiogenesis	[46]

DWI (diffusion-weighted imaging); PWI (perfusion-weighted imaging); ADC (apparent diffusion coefficient); CBF (cerebral blood flow); CBV (cerebral blood volume).

**Table 3:** Concluded Clinical Trails about Diet and Stroke.

Researchers	Year	Methods	Study Subjects	Key Findings	References
Tammy Y N Tong	2020	Personal habitual intake questionnaires	N = 418,329 European Perspective Investigation into Cancer and Nutrition Study	Higher intake of fruits, vegetables, fiber, and dairy products was associated with a reduced risk of ischemic stroke	[181]
Tina H T Chiu	2020	Food frequency questionnaires (Vegetarian status was defined by avoidance of meat and fish)	N = 13,352 1. Tzu Chi Health Study (N = 5050) 2. Tzu Chi Vegetarian Study (N = 8302)	1. Taiwanese vegetarian diet was associated with a lower risk of stroke 2. Vitamin B12 intake may modify the association between vegetarian diet and stroke	[182]
Megu Y Baden	2021	Plant-based diet index (PDI) Healthy PDI: nutritionally rich plant foods (fruit juices, refined grains) Unhealthful PDI: less nutritious plant foods (fries, sugary drinks)	N = 209,508 1. Nurses’ Health Study (NHS) 2. Health Professionals Follow-Up Study	Lower risk of total stroke was observed by those who adhered to a healthful plant-based diet	[183]
Yuan Zhang	2021	Questionnaires and interviews	N = 365,682 UK Biobank	1. Consumption of coffee and tea either separately or together was associated with reduced risks of stroke and dementia 2. Drinking coffee alone or combined with tea was also linked to lower risk of post-stroke dementia.	[192]
Daniel B Ibsen	2022	Food frequency questionnaires Assessments: EAT-Lancet diet score Alternative Healthy Eating Index-2010	N = 55,016 Danish Diet, Cancer and Health Study	1. Adherence to the EAT-Lancet diet in midlife was associated with a lower risk of subarachnoid stroke 2. AHEI (Alternate health eating index-2010) was associated with a lower risk of total stroke, mainly ischemic stroke and intracerebral hemorrhage	[186]
Andrew Smyth	2024	Beverage intake groups None, 1 to 2 cups/day, 3 to 4 cups/day, or > 4 cups/day for each beverage	N = 26,950 INTERSTROKE (a large international matched case–control study of first stroke from 32 countries)	1. High coffee consumption was associated with higher odds of ischemic stroke 2. Tea consumption was associated with lower odds of ischemic stroke	[191]
Jad El Masri	2024	DASH diet Adherence assessment: DASH diet index (ranging from 0 (lowest) to 11 (highest))	N = 428 A case-control study in Lebanese people in 2023 Ischemic cases: 214 Health controls: 214	The DASHA diet was protective against such strokes and associated with less disability	[184]
Martin J O’Donnell	2024	Food frequency questionnaires	N = 6000 INTERSTROKE	1. Carbonated beverages were linked to higher odds of stroke 2. High water intake correlated with lower stroke odds, with notable regional variations	[193]

**Table 4:** Concluded Studies about Antibiotic and Probiotics or Prebiotics Therapy.

Interventions		Study Subjects	Curative Effects
Antibiotics	Broad-spectrum antibiotic	C57BL/6J mice [197]	Broad-spectrum antibiotic reduced gut microbial populations. It did not affect brain damage within the first day post-stroke, but may suppress systemic immunity, leading to increased mortality between days 5 and 7 following stroke.
	Amoxicillin	C57BL/6J mice [48]	Gut bacterial-primed intestinal dendritic cells drive local Treg expansion, which subsequently suppresses pro-inflammatory IL-17+ γδ T cell responses.
Probiotics	Bifidobacterium longum +Lactobacillus bulgaricus +Streptococcus thermophilus	Human [211]	Daily prophylactic administration of probiotics could attenuate the deviated Th1/Th2 response induce by traumatic brain injury.
	Bidobacterium longum NK46	5xFAD mice [205]	1. Inhibited LPS-induced NF-κB activation in the colon and hippocampus; 2. Increased brain-derived neurotrophic factor expression.
	Lactobacillus salivarius	5xFAD mice [206]	1.Reduced gut leakage; Reduced the levels of IL-6 in the brain; 2. Reduced oxidative stress in the cortex and the hippocampus
Prebiotics	FOS+GOS	Human [200]	1. FOS+GOS did not affect biological markers of stress and inflammation or mental health symptoms in healthy adults; 2. FOS+GOS increased Bifidobacterium.
	Mannan oligosaccharide (MOS)	5xFAD mice [201]	1. Increased the relative abundance of butyrate-producing bacteria; 2. Increased the level of butyrate in feces and serum; 3. Reduced serum LPS level and oxidative stress in the brain.
	Xylooligosaccharides (XOS)	APP/PS1 mice [202]	1. Restored the integrity of intestinal barrier and BBB via increased expression of tight junction proteins in intestine; 2. Reduced the expression of pro-inflammatory cytokines (IL-1β and IL-6) and immunosuppressive cytokine (IL-10) in colon and hippocampus.
	β-Glucan	APP/PS1 mice [203]	1. Increased the levels of SCFAs (propionate, butyrate, and valerate) in colon; 2. Reduced microglial and astrocytic activation in hippocampus; 3. Reduced the expression of pro-inflammatory cytokines (IL-6 and IL-1β), NF-κB and NLRP3 in hippocampus and cerebral cortex.

## Abbreviations

The following abbreviations are used in this manuscript:

MGBA	microbiota–gut–brain axis
BBB	blood–brain barrier
AIS	acute ischemic stroke
HPA	hypothalamic–pituitary–adrenal
ANS	autonomic nervous system
SCFAs	short-chain fatty acids
TMAO	trimethylamine N-oxide
LPS	lipopolysaccharides
ACh	Acetylcholine
MCAO	middle cerebral artery occlusion
FMT	fecal microbiome transplantation
GPCRs	G protein-coupled receptors
GABA	Gamma-Aminobutyric Acid
AHR	aryl hydrocarbon receptor

## References

[1] Margolis K.G., Cryan J.F., Mayer E.A. (2021). The Microbiota-Gut-Brain Axis: From Motility to Mood. Gastroenterology.

[2] Han S., Cai L., Chen P., Kuang W. (2023). A study of the correlation between stroke and gut microbiota over the last 20years: A bibliometric analysis. Front. Microbiol..

[3] Katan M., Luft A. (2018). Global Burden of Stroke. Semin. Neurol..

[4] Wang T., Pan C., Xie C., Chen L., Song Z., Liao H., Xin C. (2023). Microbiota Metabolites and Immune Regulation Affect Ischemic Stroke Occurrence, Development, and Prognosis. Mol. Neurobiol..

[5] Durgan D.J., Lee J., McCullough L.D., Bryan R.M. (2019). Examining the Role of the Microbiota-Gut-Brain Axis in Stroke. Stroke.

[6] Bonkhoff A.K., Rubsamen N., Grefkes C., Rost N.S., Berger K., Karch A. (2022). Development and Validation of Prediction Models for Severe Complications After Acute Ischemic Stroke: A Study Based on the Stroke Registry of Northwestern Germany. J. Am. Heart Assoc..

[7] Xia G.H., You C., Gao X.X., Zeng X.L., Zhu J.J., Xu K.Y., Tan C.H., Xu R.T., Wu Q.H., Zhou H.W. (2019). Stroke Dysbiosis Index (SDI) in Gut Microbiome Are Associated With Brain Injury and Prognosis of Stroke. Front. Neurol..

[8] Hu W., Kong X., Wang H., Li Y., Luo Y. (2022). Ischemic stroke and intestinal flora: An insight into brain-gut axis. Eur. J. Med. Res..

[9] Zhang X., Wang X., Zhao H., Cao R., Dang Y., Yu B. (2023). Imbalance of Microbacterial Diversity Is Associated with Functional Prognosis of Stroke. Neural Plast..

[10] Sudo N., Chida Y., Aiba Y., Sonoda J., Oyama N., Yu X.N., Kubo C., Koga Y. (2004). Postnatal microbial colonization programs the hypothalamic–pituitary–adrenal system for stress response in mice. J. Physiol..

[11] Longo S., Rizza S., Federici M. (2023). Microbiota-gut-brain axis: Relationships among the vagus nerve, gut microbiota, obesity, and diabetes. Acta Diabetol..

[12] Socala K., Doboszewska U., Szopa A., Serefko A., Wlodarczyk M., Zielinska A., Poleszak E., Fichna J., Wlaz P. (2021). The role of microbiota-gut-brain axis in neuropsychiatric and neurological disorders. Pharmacol. Res..

[13] Zhao L., Yang L., Guo Y., Xiao J., Zhang J., Xu S. (2022). New Insights into Stroke Prevention and Treatment: Gut Microbiome. Cell. Mol. Neurobiol..

[14] Nakhal M.M., Yassin L.K., Alyaqoubi R., Saeed S., Alderei A., Alhammadi A., Alshehhi M., Almehairbi A., Al Houqani S., BaniYas S. (2024). The Microbiota-Gut-Brain Axis and Neurological Disorders: A Comprehensive Review. Life.

[15] Cryan J.F., O’Riordan K.J., Cowan C.S.M., Sandhu K.V., Bastiaanssen T.F.S., Boehme M., Codagnone M.G., Cussotto S., Fulling C., Golubeva A.V. (2019). The Microbiota-Gut-Brain Axis. Physiol. Rev..

[16] Thursby E., Juge N. (2017). Introduction to the human gut microbiota. Biochem. J..

[17] Ashique S., Mohanto S., Ahmed M.G., Mishra N., Garg A., Chellappan D.K., Omara T., Iqbal S., Kahwa I. (2024). Gut-brain axis: A cutting-edge approach to target neurological disorders and potential synbiotic application. Heliyon.

[18] Rose C.F., Amodio P., Bajaj J.S., Dhiman R.K., Montagnese S., Taylor-Robinson S.D., Vilstrup H., Jalan R. (2020). Hepatic encephalopathy: Novel insights into classification, pathophysiology and therapy. J. Hepatol..

[19] Yassin L.K., Nakhal M.M., Alderei A., Almehairbi A., Mydeen A.B., Akour A., Hamad M.I.K. (2025). Exploring the microbiota-gut-brain axis: Impact on brain structure and function. Front. Neuroanat..

[20] Ho J.P., Powers W.J. (2025). Contemporary Management of Acute Ischemic Stroke. Annu. Rev. Med..

[21] Scheldeman L., Wouters A., Dupont P., Christensen S., Boutitie F., Cheng B., Ebinger M., Endres M., Fiebach J.B., Gerloff C. (2021). Reversible Edema in the Penumbra Correlates With Severity of Hypoperfusion. Stroke.

[22] Bachtiar N.A., Murtala B., Muis M., Ilyas M.I., Abdul Hamid H.B., As’ad S., Tammasse J., Wuysang A.D., Soraya G.V. (2024). Non-Contrast MRI Sequences for Ischemic Stroke: A Concise Overview for Clinical Radiologists. Vasc. Health Risk Manag..

[23] Thomalla G., Simonsen C.Z., Boutitie F., Andersen G., Berthezene Y., Cheng B., Cheripelli B., Cho T.H., Fazekas F., Fiehler J. (2018). MRI-Guided Thrombolysis for Stroke with Unknown Time of Onset. N. Engl. J. Med..

[24] Feil K., Reidler P., Kunz W.G., Küpper C., Heinrich J., Laub C., Müller K., Vöglein J., Liebig T., Dieterich M. (2020). Addressing a real-life problem: Treatment with intravenous thrombolysis and mechanical thrombectomy in acute stroke patients with an extended time window beyond 4.5 h based on computed tomography perfusion imaging. Eur. J. Neurol..

[25] Nuszkiewicz J., Kukulska-Pawluczuk B., Piec K., Jarek D.J., Motolko K., Szewczyk-Golec K., Woźniak A. (2024). Intersecting Pathways: The Role of Metabolic Dysregulation, Gastrointestinal Microbiome, and Inflammation in Acute Ischemic Stroke Pathogenesis and Outcomes. J. Clin. Med..

[26] Jayaraj R.L., Azimullah S., Beiram R., Jalal F.Y., Rosenberg G.A. (2019). Neuroinflammation: Friend and foe for ischemic stroke. J. Neuroinflamm..

[27] Zeng J., Bao T., Yang K., Zhu X., Wang S., Xiang W., Ge A., Zeng L., Ge J. (2022). The mechanism of microglia-mediated immune inflammation in ischemic stroke and the role of natural botanical components in regulating microglia: A review. Front. Immunol..

[28] Li Y., Zhang J. (2021). Animal models of stroke. Anim. Model. Exp. Med..

[29] Bano N., Khan S., Ahamad S., Kanshana J.S., Dar N.J., Khan S., Nazir A., Bhat S.A. (2024). Microglia and gut microbiota: A double-edged sword in Alzheimer’s disease. Ageing Res. Rev..

[30] Li H., Liu P., Zhang B., Yuan Z., Guo M., Zou X., Qian Y., Deng S., Zhu L., Cao X. (2023). Acute ischemia induces spatially and transcriptionally distinct microglial subclusters. Genome Med..

[31] Abdel-Haq R., Schlachetzki J.C.M., Glass C.K., Mazmanian S.K. (2019). Microbiome-microglia connections via the gut-brain axis. J. Exp. Med..

[32] Kwon H.S., Koh S.H. (2020). Neuroinflammation in neurodegenerative disorders: The roles of microglia and astrocytes. Transl. Neurodegener..

[33] Ament Z., Patki A., Bhave V.M., Chaudhary N.S., Garcia Guarniz A.L., Kijpaisalratana N., Judd S.E., Cushman M., Long D.L., Irvin M.R. (2023). Gut microbiota-associated metabolites and risk of ischemic stroke in REGARDS. J. Cereb. Blood Flow Metab..

[34] Yao X., Yang C., Jia X., Yu Z., Wang C., Zhao J., Chen Y., Xie B., Zhuang H., Sun C. (2024). High-fat diet consumption promotes adolescent neurobehavioral abnormalities and hippocampal structural alterations via microglial overactivation accompanied by an elevated serum free fatty acid concentration. Brain Behav. Immun..

[35] Yang S., Miyazaki H., Wannakul T., Amo E., Saido T., Saito T., Sasaguri H., Maekawa M., Owada Y. (2025). High-Fat Diet-Induced Excessive Accumulation of Cerebral Cholesterol Esters and Microglial Dysfunction Exacerbate Alzheimer’s Disease Pathology in *APP^NL-G-F^* mice. Mol. Neurobiol..

[36] Liu X., Li X., Xia B., Jin X., Zou Q., Zeng Z., Zhao W., Yan S., Li L., Yuan S. (2021). High-fiber diet mitigates maternal obesity-induced cognitive and social dysfunction in the offspring via gut-brain axis. Cell Metab..

[37] Deczkowska A., Amit I., Schwartz M. (2018). Microglial immune checkpoint mechanisms. Nat. Neurosci..

[38] Sadler R., Cramer J.V., Heindl S., Kostidis S., Betz D., Zuurbier K.R., Northoff B.H., Heijink M., Goldberg M.P., Plautz E.J. (2020). Short-Chain Fatty Acids Improve Poststroke Recovery via Immunological Mechanisms. J. Neurosci..

[39] Fan X., Wang S., Hu S., Yang B., Zhang H. (2022). Host-microbiota interactions: The aryl hydrocarbon receptor in the acute and chronic phases of cerebral ischemia. Front. Immunol..

[40] Dong F., Perdew G.H. (2020). The aryl hydrocarbon receptor as a mediator of host-microbiota interplay. Gut Microbes.

[41] Wang M., Pan W., Xu Y., Zhang J., Wan J., Jiang H. (2022). Microglia-Mediated Neuroinflammation: A Potential Target for the Treatment of Cardiovascular Diseases. J. Inflamm. Res..

[42] Garofalo S., Cocozza G., Bernardini G., Savage J., Raspa M., Aronica E., Tremblay M.-E., Ransohoff R.M., Santoni A., Limatola C. (2022). Blocking immune cell infiltration of the central nervous system to tame Neuroinflammation in Amyotrophic lateral sclerosis. Brain Behav. Immun..

[43] Ohno H. (2016). Intestinal M cells. J. Biochem..

[44] McDermott A.J., Huffnagle G.B. (2014). The microbiome and regulation of mucosal immunity. Immunology.

[45] Malone K., Amu S., Moore A.C., Waeber C. (2019). The immune system and stroke: From current targets to future therapy. Immunol. Cell Biol..

[46] Yang S.H., Liu R. (2021). Four Decades of Ischemic Penumbra and Its Implication for Ischemic Stroke. Transl. Stroke Res..

[47] Lee J., d’Aigle J., Atadja L., Quaicoe V., Honarpisheh P., Ganesh B.P., Hassan A., Graf J., Petrosino J., Putluri N. (2020). Gut Microbiota-Derived Short-Chain Fatty Acids Promote Poststroke Recovery in Aged Mice. Circ. Res..

[48] Benakis C., Brea D., Caballero S., Faraco G., Moore J., Murphy M., Sita G., Racchumi G., Ling L., Pamer E.G. (2016). Commensal microbiota affects ischemic stroke outcome by regulating intestinal γδ T cells. Nat. Med..

[49] Zhu W., Romano K.A., Li L., Buffa J.A., Sangwan N., Prakash P., Tittle A.N., Li X.S., Fu X., Androjna C. (2021). Gut microbes impact stroke severity via the trimethylamine N-oxide pathway. Cell Host Microbe.

[50] Zeng X., Li J., Shan W., Lai Z., Zuo Z. (2023). Gut microbiota of old mice worsens neurological outcome after brain ischemia via increased valeric acid and IL-17 in the blood. Microbiome.

[51] Stuckey S.M., Ong L.K., Collins-Praino L.E., Turner R.J. (2021). Neuroinflammation as a Key Driver of Secondary Neurodegeneration Following Stroke?. Int. J. Mol. Sci..

[52] Okada T., Suzuki H., Travis Z.D., Zhang J.H. (2020). The Stroke-Induced Blood-Brain Barrier Disruption: Current Progress of Inspection Technique, Mechanism, and Therapeutic Target. Curr. Neuropharmacol..

[53] Yang Z., Wei F., Zhang B., Luo Y., Xing X., Wang M., Chen R., Sun G., Sun X. (2022). Cellular Immune Signal Exchange From Ischemic Stroke to Intestinal Lesions Through Brain-Gut Axis. Front. Immunol..

[54] Singh V., Roth S., Llovera G., Sadler R., Garzetti D., Stecher B., Dichgans M., Liesz A. (2016). Microbiota Dysbiosis Controls the Neuroinflammatory Response after Stroke. J. Neurosci..

[55] Liu F., Cheng X., Zhong S., Liu C., Jolkkonen J., Zhang X., Liang Y., Liu Z., Zhao C. (2020). Communications Between Peripheral and the Brain-Resident Immune System in Neuronal Regeneration After Stroke. Front. Immunol..

[56] Chai Z., Zheng J., Shen J. (2024). Mechanism of ferroptosis regulating ischemic stroke and pharmacologically inhibiting ferroptosis in treatment of ischemic stroke. CNS Neurosci. Ther..

[57] Fann D.Y.-W., Lee S.-Y., Manzanero S., Chunduri P., Sobey C.G., Arumugam T.V. (2013). Pathogenesis of acute stroke and the role of inflammasomes. Ageing Res. Rev..

[58] Huang Q., Xia J. (2021). Influence of the gut microbiome on inflammatory and immune response after stroke. Neurol. Sci..

[59] Yan C., Liu Z., Xie W., Zhang T., Zhang J., Li G., Xu X., Ye L., Gong J. (2024). Cornuside protects against ischemic stroke in rats by suppressing the IL-17F/TRAF6/NF-κB pathway via the brain-gut axis. Exp. Neurol..

[60] Jiang Y., Dai Y., Liu Z., Liao Y., Sun S., Kong X., Hu J., Tang Y. (2023). The role of IL-23/IL-17 axis in ischemic stroke from the perspective of gut-brain axis. Neuropharmacology.

[61] Huang A., Ji L., Li Y., Li Y., Yu Q. (2023). Gut microbiome plays a vital role in post-stroke injury repair by mediating neuroinflammation. Int. Immunopharmacol..

[62] Chen D., Xie J., Chen X., Qin B., Kong D., Luo J. (2025). Fecal microbiota transplantation alleviates neuronal Apoptosis, necroptosis and reactive microglia activation after ischemic stroke. Neuroscience.

[63] Fang Z., Chen M., Qian J., Wang C., Zhang J. (2023). The Bridge Between Ischemic Stroke and Gut Microbes: Short-Chain Fatty Acids. Cell. Mol. Neurobiol..

[64] Duan H., Hu J., Deng Y., Zou J., Ding W., Peng Q., Duan R., Sun J., Zhu J. (2023). Berberine Mediates the Production of Butyrate to Ameliorate Cerebral Ischemia via the Gut Microbiota in Mice. Nutrients.

[65] Conesa M.P.B., Blixt F.W., Peesh P., Khan R., Korf J., Lee J., Jagadeesan G., Andersohn A., Das T.K., Tan C. (2023). Stabilizing histamine release in gut mast cells mitigates peripheral and central inflammation after stroke. J. Neuroinflamm..

[66] Xu K., Gao X., Xia G., Chen M., Zeng N., Wang S., You C., Tian X., Di H., Tang W. (2021). Rapid gut dysbiosis induced by stroke exacerbates brain infarction in turn. Gut.

[67] Cui W., Xu L., Huang L., Tian Y., Yang Y., Li Y., Yu Q. (2023). Changes of gut microbiota in patients at different phases of stroke. CNS Neurosci. Ther..

[68] Li X., Wang C., Zhu J., Lin Q., Yu M., Wen J., Feng J., Hu C. (2022). Sodium Butyrate Ameliorates Oxidative Stress-Induced Intestinal Epithelium Barrier Injury and Mitochondrial Damage through AMPK-Mitophagy Pathway. Oxidative Med. Cell. Longev..

[69] Sun M., Ma N., He T., Johnston L.J., Ma X. (2020). Tryptophan (Trp) modulates gut homeostasis via aryl hydrocarbon receptor (AhR). Crit. Rev. Food Sci. Nutr..

[70] Krautkramer K.A., Fan J., Bäckhed F. (2021). Gut microbial metabolites as multi-kingdom intermediates. Nat. Rev. Microbiol..

[71] Kim C.H. (2021). Control of lymphocyte functions by gut microbiota-derived short-chain fatty acids. Cell. Mol. Immunol..

[72] Yang W., Yu T., Huang X., Bilotta A.J., Xu L., Lu Y., Sun J., Pan F., Zhou J., Zhang W. (2020). Intestinal microbiota-derived short-chain fatty acids regulation of immune cell IL-22 production and gut immunity. Nat. Commun..

[73] Zhao Y., Chen F., Wu W., Sun M., Bilotta A.J., Yao S., Xiao Y., Huang X., Eaves-Pyles T.D., Golovko G. (2018). GPR43 mediates microbiota metabolite SCFA regulation of antimicrobial peptide expression in intestinal epithelial cells via activation of mTOR and STAT3. Mucosal Immunol..

[74] van der Hee B., Wells J.M. (2021). Microbial Regulation of Host Physiology by Short-chain Fatty Acids. Trends Microbiol..

[75] Wang R., Cao S., Bashir M.E.H., Hesser L.A., Su Y., Hong S.M.C., Thompson A., Culleen E., Sabados M., Dylla N.P. (2023). Treatment of peanut allergy and colitis in mice via the intestinal release of butyrate from polymeric micelles. Nat. Biomed. Eng..

[76] Haghikia A., Zimmermann F., Schumann P., Jasina A., Roessler J., Schmidt D., Heinze P., Kaisler J., Nageswaran V., Aigner A. (2022). Propionate attenuates atherosclerosis by immune-dependent regulation of intestinal cholesterol metabolism. Eur. Heart J..

[77] Carmody R.N., Bisanz J.E. (2023). Roles of the gut microbiome in weight management. Nat. Rev. Microbiol..

[78] Palmnäs-Bédard M.S.A., Costabile G., Vetrani C., Åberg S., Hjalmarsson Y., Dicksved J., Riccardi G., Landberg R. (2022). The human gut microbiota and glucose metabolism: A scoping review of key bacteria and the potential role of SCFAs. Am. J. Clin. Nutr..

[79] Zhao L., Zhang F., Ding X., Wu G., Lam Y.Y., Wang X., Fu H., Xue X., Lu C., Ma J. (2018). Gut bacteria selectively promoted by dietary fibers alleviate type 2 diabetes. Science.

[80] Dalile B., Van Oudenhove L., Vervliet B., Verbeke K. (2019). The role of short-chain fatty acids in microbiota-gut-brain communication. Nat. Rev. Gastroenterol. Hepatol..

[81] Wong T.S., Li G., Li S., Gao W., Chen G., Gan S., Zhang M., Li H., Wu S., Du Y. (2023). G protein-coupled receptors in neurodegenerative diseases and psychiatric disorders. Signal Transduct. Target. Ther..

[82] Erny D., Hrabě de Angelis A.L., Jaitin D., Wieghofer P., Staszewski O., David E., Keren-Shaul H., Mahlakoiv T., Jakobshagen K., Buch T. (2015). Host microbiota constantly control maturation and function of microglia in the CNS. Nat. Neurosci..

[83] Caetano-Silva M.E., Rund L., Hutchinson N.T., Woods J.A., Steelman A.J., Johnson R.W. (2023). Inhibition of inflammatory microglia by dietary fiber and short-chain fatty acids. Sci. Rep..

[84] Song L., Sun Q., Zheng H., Zhang Y., Wang Y., Liu S., Duan L. (2022). Roseburia hominis Alleviates Neuroinflammation via Short-Chain Fatty Acids through Histone Deacetylase Inhibition. Mol. Nutr. Food Res..

[85] Wang H., Zhang M., Li J., Liang J., Yang M., Xia G., Ren Y., Zhou H., Wu Q., He Y. (2022). Gut microbiota is causally associated with poststroke cognitive impairment through lipopolysaccharide and butyrate. J. Neuroinflamm..

[86] Nam H.S. (2019). Gut Microbiota and Ischemic Stroke: The Role of Trimethylamine N-Oxide. J. Stroke.

[87] Connell E., Le Gall G., Pontifex M.G., Sami S., Cryan J.F., Clarke G., Müller M., Vauzour D. (2022). Microbial-derived metabolites as a risk factor of age-related cognitive decline and dementia. Mol. Neurodegener..

[88] Li Z., He X., Fang Q., Yin X. (2024). Gut Microbe-Generated Metabolite Trimethylamine-N-Oxide and Ischemic Stroke. Biomolecules.

[89] Zhang Y., Wang G., Li R., Liu R., Yu Z., Zhang Z., Wan Z. (2023). Trimethylamine N-oxide aggravated cognitive impairment from APP/PS1 mice and protective roles of voluntary exercise. Neurochem. Int..

[90] Wang Q.J., Shen Y.E., Wang X., Fu S., Zhang X., Zhang Y.N., Wang R.T. (2020). Concomitant memantine and *Lactobacillus plantarum* treatment attenuates cognitive impairments in APP/PS1 mice. Aging.

[91] Dolkar P., Deyang T., Anand N., Rathipriya A.G., Hediyal T.A., Chandrasekaran V., Krishnamoorthy N.K., Gorantla V.R., Bishir M., Rashan L. (2024). Trimethylamine-N-oxide and cerebral stroke risk: A review. Neurobiol. Dis..

[92] Nie J., Xie L., Zhao B.X., Li Y., Qiu B., Zhu F., Li G.F., He M., Wang Y., Wang B. (2018). Serum Trimethylamine N-Oxide Concentration Is Positively Associated With First Stroke in Hypertensive Patients. Stroke.

[93] Escobar C., Aldeguer X., Vivas D., Manzano Fernández S., Gonzalez Caballero E., Garcia Martín A., Barrios V., Freixa-Pamias R. (2025). The gut microbiota and its role in the development of cardiovascular disease. Expert Rev. Cardiovasc. Ther..

[94] Guo S., Bai H., Han Y., Wu Y., Peng R., Zhang X., Liang B., Zhao Q., Ma M., Zhang P. (2025). Association of the gut microbe-dependent trimethylamine N-oxide and its precursors with risk of hypertension: A cross-sectional study in rural northeastern China. Nutr. Metab. Cardiovasc. Dis..

[95] Wu C., Xue F., Lian Y., Zhang J., Wu D., Xie N., Chang W., Chen F., Wang L., Wei W. (2020). Relationship between elevated plasma trimethylamine N-oxide levels and increased stroke injury. Neurology.

[96] Zhang W., Dong X.Y., Huang R. (2023). Gut Microbiota in Ischemic Stroke: Role of Gut Bacteria-Derived Metabolites. Transl. Stroke Res..

[97] Klimiec E., Pera J., Chrzanowska-Wasko J., Golenia A., Slowik A., Dziedzic T. (2016). Plasma endotoxin activity rises during ischemic stroke and is associated with worse short-term outcome. J. Neuroimmunol..

[98] Kurita N., Yamashiro K., Kuroki T., Tanaka R., Urabe T., Ueno Y., Miyamoto N., Takanashi M., Shimura H., Inaba T. (2020). Metabolic endotoxemia promotes neuroinflammation after focal cerebral ischemia. J. Cereb. Blood Flow Metab..

[99] Needham B.D., Kaddurah-Daouk R., Mazmanian S.K. (2020). Gut microbial molecules in behavioural and neurodegenerative conditions. Nat. Rev. Neurosci..

[100] Dodd D., Spitzer M.H., Van Treuren W., Merrill B.D., Hryckowian A.J., Higginbottom S.K., Le A., Cowan T.M., Nolan G.P., Fischbach M.A. (2017). A gut bacterial pathway metabolizes aromatic amino acids into nine circulating metabolites. Nature.

[101] Agus A., Planchais J., Sokol H. (2018). Gut Microbiota Regulation of Tryptophan Metabolism in Health and Disease. Cell Host Microbe.

[102] Platten M., Nollen E.A.A., Röhrig U.F., Fallarino F., Opitz C.A. (2019). Tryptophan metabolism as a common therapeutic target in cancer, neurodegeneration and beyond. Nat. Rev. Drug Discov..

[103] Zhou M., Fan Y., Xu L., Yu Z., Wang S., Xu H., Zhang J., Zhang L., Liu W., Wu L. (2023). Microbiome and tryptophan metabolomics analysis in adolescent depression: Roles of the gut microbiota in the regulation of tryptophan-derived neurotransmitters and behaviors in human and mice. Microbiome.

[104] Teunis C.J., Stroes E.S.G., Boekholdt S.M., Wareham N.J., Murphy A.J., Nieuwdorp M., Hazen S.L., Hanssen N.M.J. (2023). Tryptophan metabolites and incident cardiovascular disease: The EPIC-Norfolk prospective population study. Atherosclerosis.

[105] Liu D., Hong Y., Chen Z., Ma Y., Xia S., Gu S., Zuo H. (2024). The Tryptophan Index Is Associated with Risk of Ischemic Stroke: A Community-Based Nested Case-Control Study. Nutrients.

[106] Gao K., Mu C.-L., Farzi A., Zhu W.-Y. (2020). Tryptophan Metabolism: A Link Between the Gut Microbiota and Brain. Adv. Nutr. Int. Rev. J..

[107] Zhou Y., Chen Y., He H., Peng M., Zeng M., Sun H. (2023). The role of the indoles in microbiota-gut-brain axis and potential therapeutic targets: A focus on human neurological and neuropsychiatric diseases. Neuropharmacology.

[108] Honarpisheh P., Lee J., Banerjee A., Korf J., Ko K.A., Blasco-Conesa M.P., Honarpisheh P., Bryan R., McCullough L., Ganesh B.P. (2022). Abstract WP240: Beneficial Gut Microbiome-Derived Ligands Can Outcompete Detrimental Brain-Derived Ligands Of Aryl Hydrocarbon Receptor After Stroke. Stroke.

[109] Marcinkowska M., Mordyl B., Siwek A., Głuch-Lutwin M., Karcz T., Gawalska A., Sapa M., Bucki A., Szafrańska K., Pomierny B. (2023). Dual Molecules Targeting 5-HT_6_ and GABA-A Receptors as a New Approach to Combat Depression Associated with Neuroinflammation. ACS Chem. Neurosci..

[110] Sharma A., Castellani R.J., Smith M.A., Muresanu D.F., Dey P.K., Sharma H.S. (2019). 5-Hydroxytryptophan: A precursor of serotonin influences regional blood-brain barrier breakdown, cerebral blood flow, brain edema formation, and neuropathology. Int. Rev. Neurobiol..

[111] Dicks L.M.T. (2022). Gut Bacteria and Neurotransmitters. Microorganisms.

[112] Sutanto C.N., Xia X., Heng C.W., Tan Y.S., Lee D.P.S., Fam J., Kim J.E. (2024). The impact of 5-hydroxytryptophan supplementation on sleep quality and gut microbiota composition in older adults: A randomized controlled trial. Clin. Nutr..

[113] Fung T.C., Vuong H.E., Luna C.D.G., Pronovost G.N., Aleksandrova A.A., Riley N.G., Vavilina A., McGinn J., Rendon T., Forrest L.R. (2019). Intestinal serotonin and fluoxetine exposure modulate bacterial colonization in the gut. Nat. Microbiol..

[114] Bruijn N., van Lohuizen R., Boron M., Fitzek M., Gabriele F., Giuliani G., Melgarejo L., Řehulka P., Sebastianelli G., Triller P. (2024). Influence of metabolic state and body composition on the action of pharmacological treatment of migraine. J. Headache Pain.

[115] Petersen C.L., Hougaard A., Gaist D., Hallas J. (2024). Risk of Stroke and Myocardial Infarction Among Initiators of Triptans. JAMA Neurol..

[116] Park C., Rosenblat J.D., Brietzke E., Pan Z., Lee Y., Cao B., Zuckerman H., Kalantarova A., McIntyre R.S. (2019). Stress, epigenetics and depression: A systematic review. Neurosci. Biobehav. Rev..

[117] Mortensen J.K., Kraglund K.L., Johnsen S.P., Mors O., Andersen G., Buttenschøn H.N. (2018). The Serotonin Transporter Gene Polymorphisms and Risk of Ischemic Stroke. Cerebrovasc. Dis..

[118] Wang D., Szyf M., Benkelfat C., Provençal N., Turecki G., Caramaschi D., Côté S.M., Vitaro F., Tremblay R.E., Booij L. (2012). Peripheral SLC6A4 DNA methylation is associated with in vivo measures of human brain serotonin synthesis and childhood physical aggression. PLoS ONE.

[119] Ansorge M.S., Zhou M., Lira A., Hen R., Gingrich J.A. (2004). Early-life blockade of the 5-HT transporter alters emotional behavior in adult mice. Science.

[120] Kang H.J., Lee E.H., Kim J.W., Kim S.W., Shin I.S., Kim J.T., Park M.S., Cho K.H., Han J.S., Lyoo I.K. (2021). Association of SLC6A4 methylation with long-term outcomes after stroke: Focus on the interaction with suicidal ideation. Sci. Rep..

[121] Troubat R., Barone P., Leman S., Desmidt T., Cressant A., Atanasova B., Brizard B., El Hage W., Surget A., Belzung C. (2021). Neuroinflammation and depression: A review. Eur. J. Neurosci..

[122] Abdou A.M., Higashiguchi S., Horie K., Kim M., Hatta H., Yokogoshi H. (2006). Relaxation and immunity enhancement effects of gamma-aminobutyric acid (GABA) administration in humans. Biofactors.

[123] Xing Y., Zhang A., Li C., Han J., Wang J., Luo L., Chang X., Tian Z., Bai Y. (2023). Corticostriatal Projections Relying on GABA Levels Mediate Exercise-Induced Functional Recovery in Cerebral Ischemic Mice. Mol. Neurobiol..

[124] Lamtahri R., Hazime M., Gowing E.K., Nagaraja R.Y., Maucotel J., Alasoadura M., Quilichini P.P., Lehongre K., Lefranc B., Gach-Janczak K. (2021). The Gliopeptide ODN, a Ligand for the Benzodiazepine Site of GABA_A_ Receptors, Boosts Functional Recovery after Stroke. J. Neurosci..

[125] Chabriat H., Bassetti C.L., Marx U., Audoli-Inthavong M.L., Sors A., Lambert E., Wattez M., Hermann D.M. (2020). Safety and efficacy of GABA_A_ α5 antagonist S44819 in patients with ischaemic stroke: A multicentre, double-blind, randomised, placebo-controlled trial. Lancet Neurol..

[126] Baj A., Moro E., Bistoletti M., Orlandi V., Crema F., Giaroni C. (2019). Glutamatergic Signaling Along The Microbiota-Gut-Brain Axis. Int. J. Mol. Sci..

[127] Janik R., Thomason L.A.M., Stanisz A.M., Forsythe P., Bienenstock J., Stanisz G.J. (2016). Magnetic resonance spectroscopy reveals oral Lactobacillus promotion of increases in brain GABA, N-acetyl aspartate and glutamate. Neuroimage.

[128] Petitfils C., Maurel S., Payros G., Hueber A., Agaiz B., Gazzo G., Marrocco R., Auvray F., Langevin G., Motta J.P. (2023). Identification of bacterial lipopeptides as key players in IBS. Gut.

[129] Olson C.A., Vuong H.E., Yano J.M., Liang Q.Y., Nusbaum D.J., Hsiao E.Y. (2018). The Gut Microbiota Mediates the Anti-Seizure Effects of the Ketogenic Diet. Cell.

[130] Wang F., Xie X., Xing X., Sun X. (2022). Excitatory Synaptic Transmission in Ischemic Stroke: A New Outlet for Classical Neuroprotective Strategies. Int. J. Mol. Sci..

[131] Andersen J.V. (2025). The Glutamate/GABA-Glutamine Cycle: Insights, Updates, and Advances. J. Neurochem..

[132] Van Harreveld A. (1959). Compounds in brain extracts causing spreading depression of cerebral cortical activity and contraction of crustacean muscle. J. Neurochem..

[133] Dawson L.A., Djali S., Gonzales C., Vinegra M.A., Zaleska M.M. (2000). Characterization of transient focal ischemia-induced increases in extracellular glutamate and aspartate in spontaneously hypertensive rats. Brain Res. Bull..

[134] Okiyama K., Smith D.H., Gennarelli T.A., Simon R.P., Leach M., McIntosh T.K. (1995). The sodium channel blocker and glutamate release inhibitor BW1003C87 and magnesium attenuate regional cerebral edema following experimental brain injury in the rat. J. Neurochem..

[135] Lai K., Pritišanac I., Liu Z.Q., Liu H.W., Gong L.N., Li M.X., Lu J.F., Qi X., Xu T.L., Forman-Kay J. (2024). Glutamate acts on acid-sensing ion channels to worsen ischaemic brain injury. Nature.

[136] Borovikova L.V., Ivanova S., Zhang M., Yang H., Botchkina G.I., Watkins L.R., Wang H., Abumrad N., Eaton J.W., Tracey K.J. (2000). Vagus nerve stimulation attenuates the systemic inflammatory response to endotoxin. Nature.

[137] Chen Z., He X., Yao M.W., Li Z., Xu X. (2021). Research advances on the cholinergic inflammatory reflex and inflammation resolution. Zhonghua Shao Shang Za Zhi.

[138] Capcha J.M.C., Rodrigues C.E., Moreira R.S., Silveira M.D., Dourado P., Dos Santos F., Irigoyen M.C., Jensen L., Garnica M.R., Noronha I.L. (2020). Wharton’s jelly-derived mesenchymal stem cells attenuate sepsis-induced organ injury partially via cholinergic anti-inflammatory pathway activation. Am. J. Physiol. Regul. Integr. Comp. Physiol..

[139] Yu L., Huang B., Po S.S., Tan T., Wang M., Zhou L., Meng G., Yuan S., Zhou X., Li X. (2017). Low-Level Tragus Stimulation for the Treatment of Ischemia and Reperfusion Injury in Patients With ST-Segment Elevation Myocardial Infarction: A Proof-of-Concept Study. JACC Cardiovasc. Interv..

[140] Zhang S., Jin M., Ren J., Sun X., Zhang Z., Luo Y., Sun X. (2023). New insight into gut microbiota and their metabolites in ischemic stroke: A promising therapeutic target. Biomed. Pharmacother..

[141] Winek K., Soreq H., Meisel A. (2021). Regulators of cholinergic signaling in disorders of the central nervous system. J. Neurochem..

[142] Jin X., Wang R.H., Wang H., Long C.L., Wang H. (2015). Brain protection against ischemic stroke using choline as a new molecular bypass treatment. Acta Pharmacol. Sin..

[143] Tan E.C.K., Johnell K., Garcia-Ptacek S., Haaksma M.L., Fastbom J., Bell J.S., Eriksdotter M. (2018). Acetylcholinesterase inhibitors and risk of stroke and death in people with dementia. Alzheimers Dement..

[144] Jiang Y., Li L., Tan X., Liu B., Zhang Y., Li C. (2015). miR-210 mediates vagus nerve stimulation-induced antioxidant stress and anti-apoptosis reactions following cerebral ischemia/reperfusion injury in rats. J. Neurochem..

[145] Ruiz A.D., Malley K.M., Danaphongse T.T., Ahmad F.N., Beltran C.M., White M.L., Baghdadi S., Pruitt D.T., Rennaker R.L., Kilgard M.P. (2023). Vagus Nerve Stimulation Must Occur During Tactile Rehabilitation to Enhance Somatosensory Recovery. Neuroscience.

[146] Schuhmann M.K., Papp L., Stoll G., Blum R., Volkmann J., Fluri F. (2021). Mesencephalic Electrical Stimulation Reduces Neuroinflammation after Photothrombotic Stroke in Rats by Targeting the Cholinergic Anti-Inflammatory Pathway. Int. J. Mol. Sci..

[147] Wu Y., Hu Y., Wang B., Li S., Ma C., Liu X., Moynagh P.N., Zhou J., Yang S. (2020). Dopamine Uses the DRD5-ARRB2-PP2A Signaling Axis to Block the TRAF6-Mediated NF-κB Pathway and Suppress Systemic Inflammation. Mol. Cell.

[148] Retzlaff C.L., Kussrow A., Schorkopf T., Saetear P., Bornhop D.J., Hardaway J.A., Sturgeon S.M., Wright J., Blakely R.D. (2017). Metallo-β-lactamase Domain-Containing Protein 1 (MBLAC1) Is a Specific, High-Affinity Target for the Glutamate Transporter Inducer Ceftriaxone. ACS Chem. Neurosci..

[149] Moody A.S., Sharma B. (2018). Multi-metal, Multi-wavelength Surface-Enhanced Raman Spectroscopy Detection of Neurotransmitters. ACS Chem. Neurosci..

[150] Gorgoraptis N., Mah Y.H., Machner B., Singh-Curry V., Malhotra P., Hadji-Michael M., Cohen D., Simister R., Nair A., Kulinskaya E. (2012). The effects of the dopamine agonist rotigotine on hemispatial neglect following stroke. Brain.

[151] Ford G.A., Bhakta B.B., Cozens A., Hartley S., Holloway I., Meads D., Pearn J., Ruddock S., Sackley C.M., Saloniki E.C. (2019). Safety and efficacy of co-careldopa as an add-on therapy to occupational and physical therapy in patients after stroke (DARS): A randomised, double-blind, placebo-controlled trial. Lancet Neurol..

[152] Chen J., Cheng M., Wang L., Zhang L., Xu D., Cao P., Wang F., Herzog H., Song S., Zhan C. (2020). A Vagal-NTS Neural Pathway that Stimulates Feeding. Curr. Biol..

[153] Borgmann D., Ciglieri E., Biglari N., Brandt C., Cremer A.L., Backes H., Tittgemeyer M., Wunderlich F.T., Bruning J.C., Fenselau H. (2021). Gut-brain communication by distinct sensory neurons differently controls feeding and glucose metabolism. Cell Metab..

[154] Cook T.M., Gavini C.K., Jesse J., Aubert G., Gornick E., Bonomo R., Gautron L., Layden B.T., Mansuy-Aubert V. (2021). Vagal neuron expression of the microbiota-derived metabolite receptor, free fatty acid receptor (FFAR3), is necessary for normal feeding behavior. Mol. Metab..

[155] Yamashiro K., Kurita N., Urabe T., Hattori N. (2021). Role of the Gut Microbiota in Stroke Pathogenesis and Potential Therapeutic Implications. Ann. Nutr. Metab..

[156] Pellegrini C., Fornai M., D’Antongiovanni V., Antonioli L., Bernardini N., Derkinderen P. (2023). The intestinal barrier in disorders of the central nervous system. Lancet Gastroenterol. Hepatol..

[157] Zhao L., Xiao J., Li S., Guo Y., Fu R., Hua S., Du Y., Xu S. (2023). The interaction between intestinal microenvironment and stroke. CNS Neurosci. Ther..

[158] Stanley D., Mason L.J., Mackin K.E., Srikhanta Y.N., Lyras D., Prakash M.D., Nurgali K., Venegas A., Hill M.D., Moore R.J. (2016). Translocation and dissemination of commensal bacteria in post-stroke infection. Nat. Med..

[159] Khan R., Di Gesù C.M., Lee J., McCullough L.D. (2024). The contribution of age-related changes in the gut-brain axis to neurological disorders. Gut Microbes.

[160] Wang D.D., Nguyen L.H., Li Y., Yan Y., Ma W., Rinott E., Ivey K.L., Shai I., Willett W.C., Hu F.B. (2021). The gut microbiome modulates the protective association between a Mediterranean diet and cardiometabolic disease risk. Nat. Med..

[161] Makki K., Deehan E.C., Walter J., Bäckhed F. (2018). The Impact of Dietary Fiber on Gut Microbiota in Host Health and Disease. Cell Host Microbe.

[162] Desai M.S., Seekatz A.M., Koropatkin N.M., Kamada N., Hickey C.A., Wolter M., Pudlo N.A., Kitamoto S., Terrapon N., Muller A. (2016). A Dietary Fiber-Deprived Gut Microbiota Degrades the Colonic Mucus Barrier and Enhances Pathogen Susceptibility. Cell.

[163] Scott S.A., Fu J., Chang P.V. (2020). Microbial tryptophan metabolites regulate gut barrier function via the aryl hydrocarbon receptor. Proc. Natl. Acad. Sci. USA.

[164] Sun J., Zhang Y., Kong Y., Ye T., Yu Q., Kumaran Satyanarayanan S., Su K.P., Liu J. (2022). Microbiota-derived metabolite Indoles induced aryl hydrocarbon receptor activation and inhibited neuroinflammation in APP/PS1 mice. Brain Behav. Immun..

[165] Terstappen G.C., Meyer A.H., Bell R.D., Zhang W. (2021). Strategies for delivering therapeutics across the blood-brain barrier. Nat. Rev. Drug Discov..

[166] Zeng M., Peng M., Liang J., Sun H. (2023). The Role of Gut Microbiota in Blood-Brain Barrier Disruption after Stroke. Mol. Neurobiol..

[167] de Rus Jacquet A., Alpaugh M., Denis H.L., Tancredi J.L., Boutin M., Decaestecker J., Beauparlant C., Herrmann L., Saint-Pierre M., Parent M. (2023). The contribution of inflammatory astrocytes to BBB impairments in a brain-chip model of Parkinson’s disease. Nat. Commun..

[168] Hoyles L., Pontifex M.G., Rodriguez-Ramiro I., Anis-Alavi M.A., Jelane K.S., Snelling T., Solito E., Fonseca S., Carvalho A.L., Carding S.R. (2021). Regulation of blood-brain barrier integrity by microbiome-associated methylamines and cognition by trimethylamine N-oxide. Microbiome.

[169] Braniste V., Al-Asmakh M., Kowal C., Anuar F., Abbaspour A., Tóth M., Korecka A., Bakocevic N., Ng L.G., Kundu P. (2014). The gut microbiota influences blood-brain barrier permeability in mice. Sci. Transl. Med..

[170] Hoyles L., Snelling T., Umlai U.K., Nicholson J.K., Carding S.R., Glen R.C., McArthur S. (2018). Microbiome-host systems interactions: Protective effects of propionate upon the blood-brain barrier. Microbiome.

[171] Stachulski A.V., Knausenberger T.B., Shah S.N., Hoyles L., McArthur S. (2023). A host-gut microbial amino acid co-metabolite, p-cresol glucuronide, promotes blood-brain barrier integrity in vivo. Tissue Barriers.

[172] Sun N., Hu H., Wang F., Li L., Zhu W., Shen Y., Xiu J., Xu Q. (2021). Antibiotic-induced microbiome depletion in adult mice disrupts blood-brain barrier and facilitates brain infiltration of monocytes after bone-marrow transplantation. Brain Behav. Immun..

[173] Brunt V.E., LaRocca T.J., Bazzoni A.E., Sapinsley Z.J., Miyamoto-Ditmon J., Gioscia-Ryan R.A., Neilson A.P., Link C.D., Seals D.R. (2021). The gut microbiome-derived metabolite trimethylamine N-oxide modulates neuroinflammation and cognitive function with aging. Geroscience.

[174] Qiao C.M., Quan W., Zhou Y., Niu G.Y., Hong H., Wu J., Zhao L.P., Li T., Cui C., Zhao W.J. (2023). Orally Induced High Serum Level of Trimethylamine N-oxide Worsened Glial Reaction and Neuroinflammation on MPTP-Induced Acute Parkinson’s Disease Model Mice. Mol. Neurobiol..

[175] Quan W., Qiao C.M., Niu G.Y., Wu J., Zhao L.P., Cui C., Zhao W.J., Shen Y.Q. (2023). Trimethylamine N-Oxide Exacerbates Neuroinflammation and Motor Dysfunction in an Acute MPTP Mice Model of Parkinson’s Disease. Brain Sci..

[176] Li D., Ke Y., Zhan R., Liu C., Zhao M., Zeng A., Shi X., Ji L., Cheng S., Pan B. (2018). Trimethylamine-N-oxide promotes brain aging and cognitive impairment in mice. Aging Cell.

[177] Deng Y., Zou J., Hong Y., Peng Q., Fu X., Duan R., Chen J., Chen X. (2022). Higher Circulating Trimethylamine N-Oxide Aggravates Cognitive Impairment Probably via Downregulating Hippocampal SIRT1 in Vascular Dementia Rats. Cells.

[178] Zhou S., Liu J., Sun Y., Xu P., Liu J.L., Sun S., Zhu B., Wu H. (2023). Dietary choline metabolite TMAO impairs cognitive function and induces hippocampal synaptic plasticity declining through the mTOR/P70S6K/4EBP1 pathway. Food Funct..

[179] Govindarajulu M., Pinky P.D., Steinke I., Bloemer J., Ramesh S., Kariharan T., Rella R.T., Bhattacharya S., Dhanasekaran M., Suppiramaniam V. (2020). Gut Metabolite TMAO Induces Synaptic Plasticity Deficits by Promoting Endoplasmic Reticulum Stress. Front. Mol. Neurosci..

[180] Tan B.Y.Q., Paliwal P.R., Sharma V.K. (2020). Gut Microbiota and Stroke. Ann. Indian Acad. Neurol..

[181] Tong T.Y.N., Appleby P.N., Key T.J., Dahm C.C., Overvad K., Olsen A., Tjonneland A., Katzke V., Kuhn T., Boeing H. (2020). The associations of major foods and fibre with risks of ischaemic and haemorrhagic stroke: A prospective study of 418 329 participants in the EPIC cohort across nine European countries. Eur. Heart J..

[182] Chiu T.H.T., Chang H.R., Wang L.Y., Chang C.C., Lin M.N., Lin C.L. (2020). Vegetarian diet and incidence of total, ischemic, and hemorrhagic stroke in 2 cohorts in Taiwan. Neurology.

[183] Baden M.Y., Shan Z., Wang F., Li Y., Manson J.E., Rimm E.B., Willett W.C., Hu F.B., Rexrode K.M. (2021). Quality of Plant-Based Diet and Risk of Total, Ischemic, and Hemorrhagic Stroke. Neurology.

[184] El Masri J., Finge H., Baroud T., Ajaj N., Houmani M., Ghazi M., Younes M., Salameh P., Hosseini H. (2024). Adherence to Dietary Approaches to Stop Hypertension (DASH) Diet as a Protective Factor for Ischemic Stroke and Its Influence on Disability Level: A Case-Control Study in Lebanon. Nutrients.

[185] Challa H.J., Ameer M.A., Uppaluri K.R. (2024). DASH Diet to Stop Hypertension. StatPearls.

[186] Ibsen D.B., Christiansen A.H., Olsen A., Tjonneland A., Overvad K., Wolk A., Mortensen J.K., Dahm C.C. (2022). Adherence to the EAT-Lancet Diet and Risk of Stroke and Stroke Subtypes: A Cohort Study. Stroke.

[187] Duan Y., Zeng L., Zheng C., Song B., Li F., Kong X., Xu K. (2018). Inflammatory Links Between High Fat Diets and Diseases. Front. Immunol..

[188] Wang X.L., Li L. (2021). Microglia Regulate Neuronal Circuits in Homeostatic and High-Fat Diet-Induced Inflammatory Conditions. Front. Cell. Neurosci..

[189] Monda A., La Torre M.E., Messina A., Di Maio G., Monda V., Moscatelli F., De Stefano M., La Marra M., Padova M.D., Dipace A. (2024). Exploring the ketogenic diet’s potential in reducing neuroinflammation and modulating immune responses. Front. Immunol..

[190] Morris G., Puri B.K., Maes M., Olive L., Berk M., Carvalho A.F. (2020). The role of microglia in neuroprogressive disorders: Mechanisms and possible neurotherapeutic effects of induced ketosis. Prog. Neuropsychopharmacol. Biol. Psychiatry.

[191] Smyth A., Hankey G.J., Langhorne P., Reddin C., Ryglewicz D., Rosengren A., Xavier D., Canavan M., Oveisgharan S., Wang X. (2024). Tea and coffee consumption and risk of acute stroke: The INTERSTROKE Study. Int. J. Stroke.

[192] Zhang Y., Yang H., Li S., Li W.D., Wang Y. (2021). Consumption of coffee and tea and risk of developing stroke, dementia, and poststroke dementia: A cohort study in the UK Biobank. PLoS Med..

[193] Smyth A., Hankey G.J., Damasceno A., Iversen H.K., Oveisgharan S., Alhussain F., Langhorne P., Xavier D., Jaramillo P.L., Oguz A. (2024). Carbonated Beverage, Fruit Drink, and Water Consumption and Risk of Acute Stroke: The INTERSTROKE Case-Control Study. J. Stroke.

[194] Jin Y., Wu Y., Zeng Z., Jin C., Wu S., Wang Y., Fu Z. (2016). From the Cover: Exposure to Oral Antibiotics Induces Gut Microbiota Dysbiosis Associated with Lipid Metabolism Dysfunction and Low-Grade Inflammation in Mice. Toxicol. Sci..

[195] Francino M.P. (2015). Antibiotics and the Human Gut Microbiome: Dysbioses and Accumulation of Resistances. Front. Microbiol..

[196] Nakajima M., Arimatsu K., Kato T., Matsuda Y., Minagawa T., Takahashi N., Ohno H., Yamazaki K. (2015). Oral Administration of *P. gingivalis* Induces Dysbiosis of Gut Microbiota and Impaired Barrier Function Leading to Dissemination of Enterobacteria to the Liver. PLoS ONE.

[197] Winek K., Engel O., Koduah P., Heimesaat M.M., Fischer A., Bereswill S., Dames C., Kershaw O., Gruber A.D., Curato C. (2016). Depletion of Cultivatable Gut Microbiota by Broad-Spectrum Antibiotic Pretreatment Worsens Outcome After Murine Stroke. Stroke.

[198] Bindels L.B., Delzenne N.M., Cani P.D., Walter J. (2015). Towards a more comprehensive concept for prebiotics. Nat. Rev. Gastroenterol. Hepatol..

[199] Gibson G.R., Hutkins R., Sanders M.E., Prescott S.L., Reimer R.A., Salminen S.J., Scott K., Stanton C., Swanson K.S., Cani P.D. (2017). Expert consensus document: The International Scientific Association for Probiotics and Prebiotics (ISAPP) consensus statement on the definition and scope of prebiotics. Nat. Rev. Gastroenterol. Hepatol..

[200] Mysonhimer A.R., Cannavale C.N., Bailey M.A., Khan N.A., Holscher H.D. (2023). Prebiotic Consumption Alters Microbiota but Not Biological Markers of Stress and Inflammation or Mental Health Symptoms in Healthy Adults: A Randomized, Controlled, Crossover Trial. J. Nutr..

[201] Liu Q., Xi Y., Wang Q., Liu J., Li P., Meng X., Liu K., Chen W., Liu X., Liu Z. (2021). Mannan oligosaccharide attenuates cognitive and behavioral disorders in the 5xFAD Alzheimer’s disease mouse model via regulating the gut microbiota-brain axis. Brain Behav. Immun..

[202] Han D., Li Z., Liu T., Yang N., Li Y., He J., Qian M., Kuang Z., Zhang W., Ni C. (2020). Prebiotics Regulation of Intestinal Microbiota Attenuates Cognitive Dysfunction Induced by Surgery Stimulation in APP/PS1 Mice. Aging Dis..

[203] Zhang Q., Zhao W., Hou Y., Song X., Yu H., Tan J., Zhou Y., Zhang H.T. (2023). β-Glucan attenuates cognitive impairment of APP/PS1 mice via regulating intestinal flora and its metabolites. CNS Neurosci. Ther..

[204] Zou X., Wang L., Xiao L., Wang S., Zhang L. (2022). Gut microbes in cerebrovascular diseases: Gut flora imbalance, potential impact mechanisms and promising treatment strategies. Front. Immunol..

[205] Lee H.J., Lee K.E., Kim J.K., Kim D.H. (2019). Suppression of gut dysbiosis by Bifidobacterium longum alleviates cognitive decline in 5XFAD transgenic and aged mice. Sci. Rep..

[206] Chen C., Ahn E.H., Kang S.S., Liu X., Alam A., Ye K. (2020). Gut dysbiosis contributes to amyloid pathology, associated with C/EBPβ/AEP signaling activation in Alzheimer’s disease mouse model. Sci. Adv..

[207] Zeng X., Gao X., Peng Y., Wu Q., Zhu J., Tan C., Xia G., You C., Xu R., Pan S. (2019). Higher Risk of Stroke Is Correlated With Increased Opportunistic Pathogen Load and Reduced Levels of Butyrate-Producing Bacteria in the Gut. Front. Cell. Infect. Microbiol..

[208] Mendelson S.J., Prabhakaran S. (2021). Diagnosis and Management of Transient Ischemic Attack and Acute Ischemic Stroke: A Review. JAMA.

[209] Chen X., Hu Y., Yuan X., Yang J., Li K. (2022). Effect of early enteral nutrition combined with probiotics in patients with stroke: A meta-analysis of randomized controlled trials. Eur. J. Clin. Nutr..

[210] Guha D., Banerjee A., Mukherjee R., Pradhan B., Peneva M., Aleksandrov G., Suklabaidya S., Senapati S., Aich P. (2019). A probiotic formulation containing Lactobacillus bulgaricus DWT1 inhibits tumor growth by activating pro-inflammatory responses in macrophages. J. Funct. Foods.

[211] Tan M., Zhu J.C., Du J., Zhang L.M., Yin H.H. (2011). Effects of probiotics on serum levels of Th1/Th2 cytokine and clinical outcomes in severe traumatic brain-injured patients: A prospective randomized pilot study. Crit. Care.

[212] Castro-Herrera V.M., Fisk H.L., Wootton M., Lown M., Owen-Jones E., Lau M., Lowe R., Hood K., Gillespie D., Hobbs F.D.R. (2021). Combination of the Probiotics Lacticaseibacillus rhamnosus GG and Bifidobacterium animalis subsp. lactis, BB-12 Has Limited Effect on Biomarkers of Immunity and Inflammation in Older People Resident in Care Homes: Results From the Probiotics to Reduce Infections iN CarE home reSidentS Randomized, Controlled Trial. Front. Immunol..

[213] Loh J.S., Mak W.Q., Tan L.K.S., Ng C.X., Chan H.H., Yeow S.H., Foo J.B., Ong Y.S., How C.W., Khaw K.Y. (2024). Microbiota–gut–brain axis and its therapeutic applications in neurodegenerative diseases. Signal Transduct. Target. Ther..

[214] Kang Y., Cai Y. (2017). Gut microbiota and obesity: Implications for fecal microbiota transplantation therapy. Hormones.

[215] Blackwood B.P., Yuan C.Y., Wood D.R., Nicolas J.D., Grothaus J.S., Hunter C.J. (2017). Probiotic Lactobacillus Species Strengthen Intestinal Barrier Function and Tight Junction Integrity in Experimental Necrotizing Enterocolitis. J. Probiotics Health.

[216] Wade H., Pan K., Duan Q., Kaluzny S., Pandey E., Fatumoju L., Saraswathi V., Wu R., Harris E.N., Su Q. (2023). Akkermansia muciniphila and its membrane protein ameliorates intestinal inflammatory stress and promotes epithelial wound healing via CREBH and miR-143/145. J. Biomed. Sci..

[217] He K.Y., Lei X.Y., Wu D.H., Zhang L., Li J.Q., Li Q.T., Yin W.T., Zhao Z.L., Liu H., Xiang X.Y. (2023). Akkermansia muciniphila protects the intestine from irradiation-induced injury by secretion of propionic acid. Gut Microbes.

[218] Mancini N.L., Rajeev S., Jayme T.S., Wang A., Keita Å.V., Workentine M.L., Hamed S., Söderholm J.D., Lopes F., Shutt T.E. (2021). Crohn’s Disease Pathobiont Adherent-Invasive E coli Disrupts Epithelial Mitochondrial Networks With Implications for Gut Permeability. Cell Mol. Gastroenterol. Hepatol..

[219] Bustamante P., Ramos-Corominas M.N., Martinez-Medina M. (2024). Contribution of Toxin-Antitoxin Systems to Adherent-Invasive *E. coli* Pathogenesis. Microorganisms.

[220] Yin J., Liao S.X., He Y., Wang S., Xia G.H., Liu F.T., Zhu J.J., You C., Chen Q., Zhou L. (2015). Dysbiosis of Gut Microbiota With Reduced Trimethylamine-N-Oxide Level in Patients With Large-Artery Atherosclerotic Stroke or Transient Ischemic Attack. J. Am. Heart Assoc..

[221] Ji W., Zhu Y., Kan P., Cai Y., Wang Z., Wu Z., Yang P. (2017). Analysis of intestinal microbial communities of cerebral infarction and ischemia patients based on high throughput sequencing technology and glucose and lipid metabolism. Mol. Med. Rep..

[222] Xu N., Kan P., Yao X., Yang P., Wang J., Xiang L., Zhu Y. (2018). Astragaloside IV reversed the autophagy and oxidative stress induced by the intestinal microbiota of AIS in mice. J. Microbiol..

[223] Wang Z., Xu K., Zhou H. (2021). Characteristics of gut virome and microbiome in patients with stroke. Nan Fang. Yi Ke Da Xue Xue Bao.

[224] Li N., Wang X., Sun C., Wu X., Lu M., Si Y., Ye X., Wang T., Yu X., Zhao X. (2019). Change of intestinal microbiota in cerebral ischemic stroke patients. BMC Microbiol..

[225] Vendrik K.E.W., Ooijevaar R.E., de Jong P.R.C., Laman J.D., van Oosten B.W., van Hilten J.J., Ducarmon Q.R., Keller J.J., Kuijper E.J., Contarino M.F. (2020). Fecal Microbiota Transplantation in Neurological Disorders. Front. Cell. Infect. Microbiol..

